# Single‐Nuclei Resolution of Intermuscular Adipose Tissue Indicates an Inflammation‐Associated Cellular Profile in Individuals With Knee Osteoarthritis: Findings From the SOMMA KOA Ancillary Study

**DOI:** 10.1111/acel.70348

**Published:** 2025-12-26

**Authors:** Line O. Elingaard‐Larsen, Katie L. Whytock, Adeline Divoux, Cheehoon Ahn, Giovanna Distefano, Bret H. Goodpaster, Paul M. Coen, Jamie N. Justice, Erin E. Kershaw, Nancy E. Lane, Lauren M. Sparks

**Affiliations:** ^1^ Translational Research Institute, AdventHealth Orlando Orlando Florida USA; ^2^ Steno Diabetes Center Copenhagen Herlev Denmark; ^3^ Department of Medicine, Division of Geriatric Medicine Wake Forest University School of Medicine Winston‐Salem North Carolina USA; ^4^ Department of Internal Medicine University of Pittsburgh Pittsburgh Pennsylvania USA; ^5^ Department of Medicine, Division of Rheumatology and Clinical Allergy U.C. Davis Health Sacramento California USA

**Keywords:** intermuscular adipose tissue, knee osteoarthritis, single nuclei RNA‐sequencing

## Abstract

Individuals with knee osteoarthritis (KOA) have skeletal muscle changes around the knee joint including reduced quadricep muscle mass and increased intermuscular adipose tissue (IMAT). We examined the cellular composition and transcriptional profiles using single‐nuclei RNA sequencing in IMAT from 6 older women with KOA and knee pain and 5 older women without KOA or knee pain from the Study of Muscle, Mobility and Aging (SOMMA). From the resulting 21,436 nuclei, we identified 6 major cell types with unique transcriptional profiles, including progenitor cells, adipocytes, macrophages and other immune cells (T/B/NK cells), endothelial cells and smooth muscle cells/pericytes. Sub‐clustering of the immune cell population revealed the presence of mast cells and B‐cells with greater abundances in the KOA group. The adipocyte population was the most transcriptional diverse population between the KOA group and the group without KOA. Cell–cell communication network analysis highlighted that adipocytes had the most prominent signaling role of all cell types, independent of KOA status; however, signaling of the pro‐inflammatory adipokine leptin was enriched in the KOA group. This study provides the first interrogation of the cellular diversity and transcriptional profiles of IMAT in individuals with KOA. Our findings suggest that IMAT may contribute to KOA disease burden potentially through pro‐inflammatory signaling.

## Graphical abstract

Interrogation of the cellular and transcriptional profile of IMAT in individuals with KOA reveals a pro‐inflammatory phenotype.

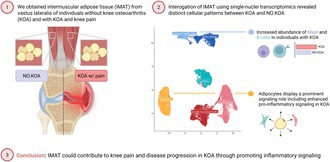

## 1 Introduction

Intermuscular adipose tissue (IMAT) is a unique adipose depot located beneath the fascia and between skeletal muscle groups, thereby enabling direct crosstalk between muscle and adipose tissue (Goodpaster et al. 2023). Deposition of IMAT generally increases with age and adiposity (Addison et al. 2014; Marcus et al. 2010; Sparks et al. 2021), as well as with type 2 diabetes and insulin resistance (Boettcher et al. 2009; Goodpaster et al. 2000, 2003). IMAT is also commonly observed in pathological conditions related to muscle degeneration and disuse (Addison et al. 2014; Goodpaster et al. 2001, 2008) and can affect muscle function causing mobility decline, especially in older adults (Addison et al. 2014).

A total of 364.6 million people are diagnosed with knee osteoarthritis (KOA) worldwide (Long et al. 2022), and the prevalence increase with age, obesity and female sex (Katz et al. 2021). Individuals with KOA have skeletal muscle changes around the knee joint including reduced quadricep muscle mass and increased IMAT quantified by magnetic resonance imaging (MRI) (Cross et al. 2014; Kumar et al. 2014, 2021; Pedroso et al. 2019). We recently reported that the presence and severity of KOA is associated with greater IMAT mass and lower leg power in older individuals from the Study of Muscle, Mobility and Aging (SOMMA) (Distefano et al. 2024). Not all individuals with KOA experience pain, but IMAT quantity has been significantly and positively associated with knee pain in women with KOA (Dannhauer et al. 2015), suggesting that the mere presence of IMAT—whether it be the mass or the molecular profile—may induce pain in the knee joint. Conversely, knee pain itself can also cause disuse and immobility leading to accumulation of IMAT.

To date, most studies have focused on IMAT quantity in relation to different disease stages. Fat mass per se, however, is not the only cause of adiposity‐driven KOA and pain. Recent studies in older individuals have demonstrated that a pro‐inflammatory profile, cellular senescence, and metabolic inflexibility develop with age in abdominal subcutaneous adipose tissue (ASAT), thus impacting its overall quality. In addition, senescent cell burden in abdominal adipose tissue parallels slower walking speed in older adults (Justice et al. 2018), highlighting the link between adipose tissue *quality* and physical function. In the case of IMAT, it is possible that the adipocytes and other cells within the IMAT depot may influence the local skeletal muscle tissue milieu and the knee joint through the delivery of factors such as cytokines that may stimulate pain through nociceptors (Eichwald and Talbot 2020).

The cellular composition of human adipose tissue depots such as ASAT is well‐established, with adipocytes comprising ~90% of total tissue volume but only ~50% of total cell number (Corvera 2021; Whytock et al. 2022, 2024). However, cell composition and their proportions remain unknown for human IMAT. We recently established a single‐nuclei RNA sequencing (snRNA‐seq) method optimized for IMAT, in which we were able to detect all major cell types (Elingaard‐Larsen et al. 2024), making it ideal for deeper interrogation of IMAT in the context of KOA.

We hypothesized that the cellular landscape and the transcriptional profile of IMAT would differ among women with KOA and pain compared to women without KOA or pain, and that the cellular composition may be associated with skeletal muscle strength and mobility. To test this hypothesis, we used snRNA‐seq to evaluate the cellular heterogeneity of IMAT in a subset of older women from the SOMMA cohort with KOA and pain and women without KOA or pain.

## 2 Results

To explore the cellular landscape of human IMAT in relation to KOA, we collected IMAT during skeletal muscle biopsies from 6 women with KOA and 5 women without KOA (NO.KOA) as part of their participation in SOMMA (Cummings et al. 2023). We selected women based on having a Kellgren‐Lawrence score between 0 and 1 (NO.KOA) or 3 and 4 (KOA). The women with KOA experienced knee pain as previously defined (Conroy et al. 2012), whereas the women without KOA did not experience knee pain. The two groups did not differ significantly in anthropometric measures including weight, waist circumference and BMI (Table 1). Based on the notion that IMAT has the potential to impact muscle function, we assessed body composition and skeletal muscle strength and mobility (Table 2). No significant differences were observed in the quantity of fat infiltration in the right thigh muscle measured with MRI between the KOA and NO.KOA groups. However, this is likely due to the small sample size in the current study, as a clear relationship between increased muscle fat infiltration and severity of KOA has been established in a larger population of older individuals with KOA in the SOMMA study (Distefano et al. 2024) and in the Health ABC study (Conroy et al. 2012). We also examined the levels of circulating immune cells in the two groups, due to the known inflammatory phenotype of KOA. White blood cells, in particular neutrophils, were elevated in the KOA group but did not reach statistical significance (*p* = 0.13 and 0.16, respectively, Table 2).

**TABLE 1:** Clinical characteristics of study participants.

	KOA	NO.KOA	*p* value
N	6	5	
Kellgren‐Lawrence score	3–4	0–1	
Knee pain			
Daily (*n*)	3	0	
Weekly (*n*)	1	0	
Monthly (*n*)	1	0	
Never	1	5	
Age (years)	79.0 ± 8.99	76.2 ± 5.97	0.55
Women (%)	100	100	
Race			
White	5	3	
Black	1	1	
Asian	0	1	
Weight (kg)	61.38 ± 7.52	62.04 ± 8.34	0.9
BMI (kg/m^2^)	24.60 ± 1.97	24.06 ± 1.31	0.6
Waist (cm)	83.68 ± 8.86	84.66 ± 11.31	0.88
SBP (mmHg)	132.83 ± 11.53	138.00 ± 20.35	0.63
DBP (mmHg)	73.33 ± 4.50	80.80 ± 8.44	0.13
HbA1c (%)	5.55 ± 0.39	5.50 ± 0.16	0.8

*Note:* Values represent mean ± standard deviation. *p* value reflects significance from Welch's *t*‐test.

Abbreviations: BMI, Body Mass Index; DBP, diastolic blood pressure; HbA1c, hemoglobin A1C; SBP, systolic blood pressure.

**TABLE 2:** Measures of body composition, muscle function, and circulating immune cells.

	KOA	NO.KOA	*p* value
Body composition			
ASAT vol, L	6.50 ± 1.65	7.88 ± 5.05	0.25
VAT vol, L	2.83 ± 1.42	2.60 ± 0.94	0.75
Total AT vol (ASAT + VAT)	9.33 ± 2.58	10.47 ± 2.21	0.46
Anterior thigh muscle fat infiltration (%)^a^	0.09 ± 0.02	0.09 ± 0.01	0.95
D3‐Cr muscle mass (kg)	17.78 ± 4.28	16.62 ± 3.60	0.64
Total thigh fat free muscle vol (L)	6.79 ± 0.87	6.56 ± 1.18	0.73
Anterior thigh fat free muscle vol (L)^a^	1.07 ± 0.19	1.11 ± 0.19	0.45
Strength and mobility			
Steps per day	6543 ± 1557	7621 ± 1340	0.34
SPPB score, 400 m walk	10.50 ± 1.87	8.80 ± 1.64	0.15
Walking speed, 400 m (m/s)	1.08 ± 0.17	1.09 ± 0.12	0.94
Stair climbing test, ascent time (s)	3.97 ± 0.89	4.56 ± 1.12	0.38
Stair climbing test, descent time (s)	5.10 ± 2.48	5.07 ± 1.20	0.98
Knee extensor peak power (watts)	244.92 ± 100.34	267.90 ± 70.51	0.68
Standardized peak power (watts/kg)	3.91 ± 1.25	4.35 ± 1.19	0.57
Specific torque (watt/thigh muscle vol)^b^	225.69 ± 57.68	242.22 ± 49.72	0.63
Circulating immune cells			
WBC (×10e3/μL)	8.23 ± 3.97	5.20 ± 0.98	0.13
Lymphocytes (×10e3/μL)	3.38 ± 3.30	1.56 ± 0.38	0.25
Monocytes (×10e3/μL)	0.57 ± 0.22	0.52 ± 0.13	0.68
Immature Granulocytes (×10e3/μL)	0.02 ± 0.04	0.00 ± 0.00	0.35
Neutrophils (×10e3/μL)	4.03 ± 1.43	2.94 ± 0.79	0.16
Basophils (×10e3/μL)	0.08 ± 0.04	0.08 ± 0.04	0.90
Eosinophils (×10e3/μL)	0.22 ± 0.12	0.14 ± 0.09	0.26

*Note:* Values represent mean ± standard deviation. *p* value reflects significance from Welch's *t*‐test.

Abbreviations: ASAT, abdominal subcutaneous adipose tissue; AT, adipose tissue; D3‐Cr, D3‐creatine; MRI, magnetic resonance imaging; SPPB, short physical performance battery; VAT, visceral adipose tissue; WBC; white blood cells.

^a^ Right anterior thigh.

^b^ Specific torque was calculated using Knee extensor Peak Power divided by Anterior thigh fat free muscle vol of the leg tested (Right/Left).

### 2.1 The Cellular Composition of IMAT

We next examined the transcriptional profile of IMAT at single‐cell resolution. We performed snRNA‐seq using a customized protocol to isolate nuclei from IMAT (Elingaard‐Larsen et al. 2024). We obtained a total of 21,436 nuclei, encompassing 10,877 nuclei from KOA individuals (*n* = 6) and 10,559 nuclei from NO.KOA individuals (*n* = 5), with an average of 1388 genes detected per nuclei. Integrating the data from all 11 participants, we detected 9 cell populations based on common gene markers and differentially expressed genes (DEGs), including three myonuclei clusters (Figure S1A,B). The top five DEGs for each cell type cluster are displayed in Figure S2A. While IMAT was carefully dissected from skeletal muscle tissue under a microscope, we consider the presence of the myogenic clusters as contamination from the muscle biopsy due to the proximity of the two tissues when sampling. Furthermore, as evident in Figure S1C, the abundance of myonuclei clusters was quite variable between participants, consequently these three clusters were excluded from the subsequent IMAT‐focused analysis.

**FIGURE 1:**
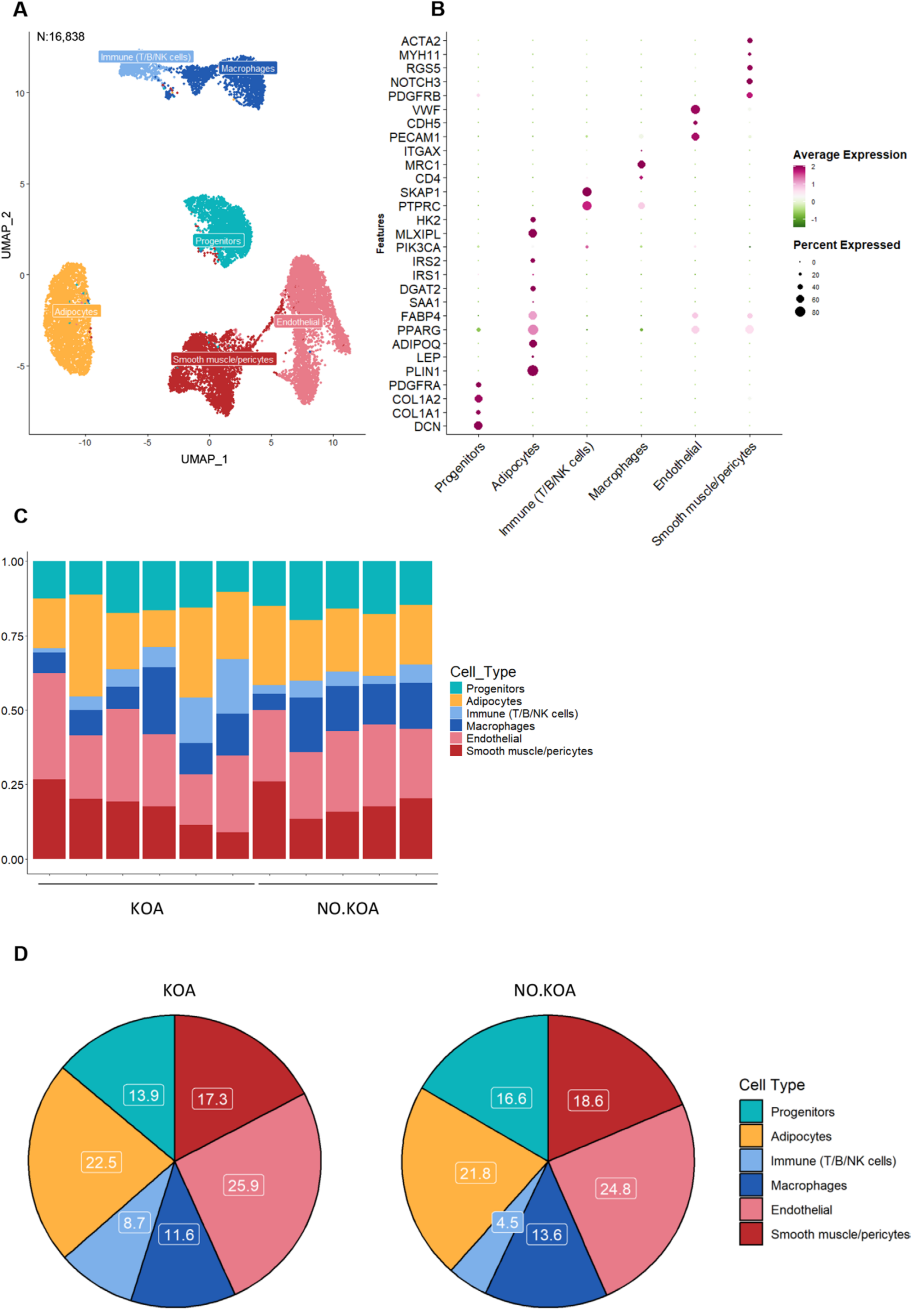
Cell type composition and proportion in IMAT. (A) UMAP depicting the six cell type clusters identified based on 16,838 nuclei from in total 11 IMAT samples. Each dot represents a nucleus. N refers to the total number of nuclei used for clustering (B) Dot plot displaying common gene markers for each of the six cell type clusters. Dot size represents the percentage of cells in the cluster which express the specific gene marker, whereas the color of the dot represents the average expression of the gene marker relative to the other cell type clusters. (C) Stacked bar plot demonstrating the proportion of each of the identified cell types for each study participant ordered according to group. (D) Pie charts showing the percentage of each of the cell types within the two groups. No statistically significant differences were found in the cell type proportion between the two groups, when tested using the linear modeling framework of the *propeller* function in the R package *speckle*.

In our non‐myogenic dataset of 16,838 nuclei (8285 KOA nuclei, 8553 NO.KOA nuclei), we identified a progenitor cell population (*PDGFRA, DCN*), adipocytes (*PLIN1*, *ADIPOQ*), macrophages (*MRC1*, *ITGAX*) and other immune cells (*PTPRC*, *SKAP1*), endothelial cells (*PECAM1*, *VWF*) and smooth muscle/pericytes (*PDGFRB*, *NOTCH3*, *MYH11*, *ACTA2*) (Figure 1A,B). The top five DEGs for each cell type cluster are displayed in Figure S2B. Importantly, each of the seven cell types was quantified in all participants (Figure 1C). Adipocytes comprised ~22% of the total nuclei, which is lower than previous reports in ASAT (Whytock et al. 2022, 2024) but in agreement with other single‐nuclei studies of ASAT and visceral adipose tissue (VAT) (Emont et al. 2022; Massier et al. 2023; Strieder‐Barboza et al. 2022), and substantially higher than a previous snRNA‐seq analysis of muscle with fatty infiltration which showed that adipocytes only comprised ~3% (Fitzgerald et al. 2023) (Figure 1D). Endothelial cells comprised ~25%, followed by smooth muscle cells and pericytes at ~18%, progenitor cells at ~15%, macrophages at ~13% and immune cells at ~7%. The proportions of the cell populations did not differ significantly between the KOA and NO.KOA groups (Figure 1D).

**FIGURE 2:**
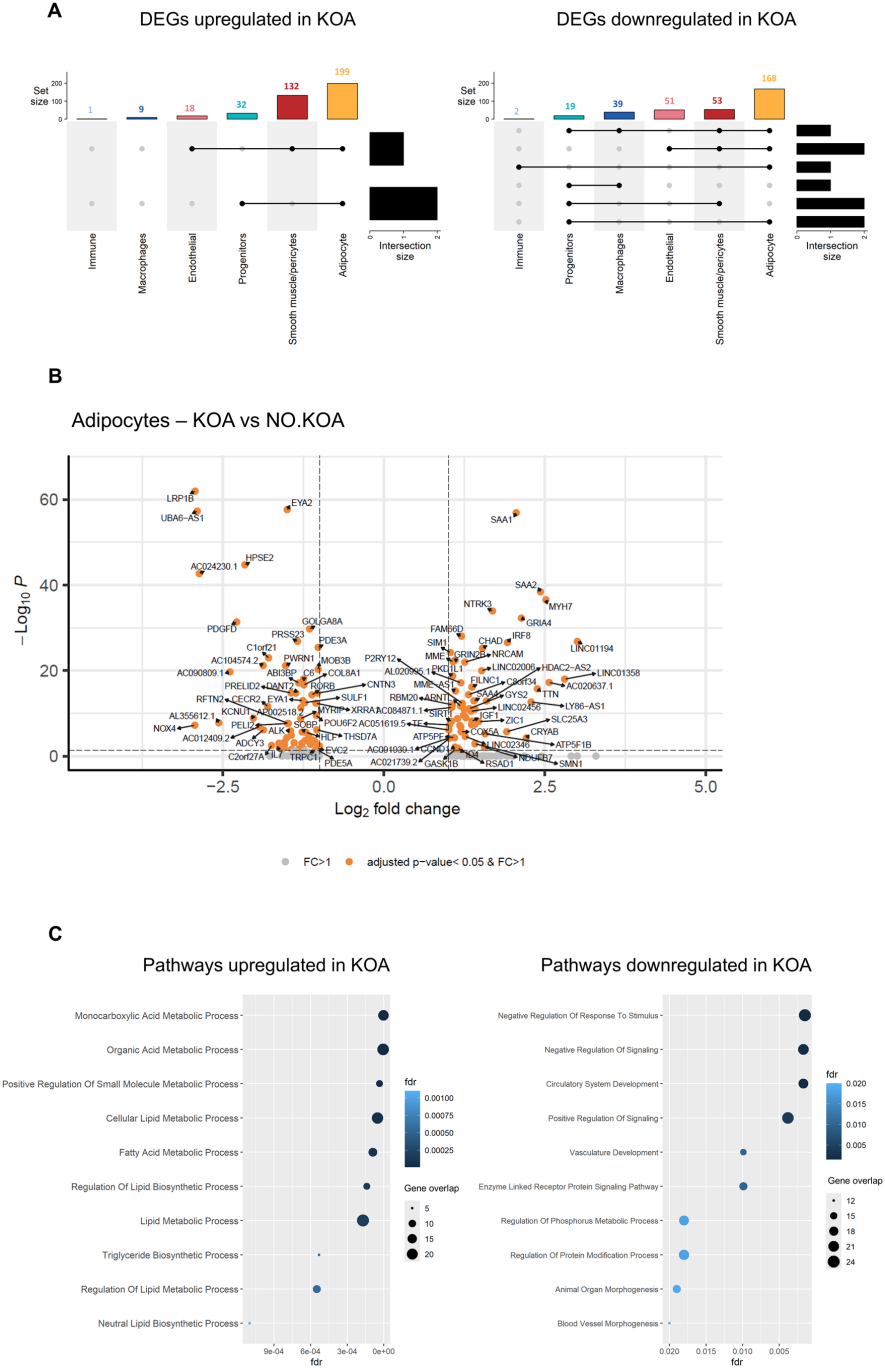
Differential gene expression and pathway analysis. (A) UpSet plot displaying the number of genes differentially expressed within each cell type (FDR < 0.05 and −0.5 < log2FC > 0.5) separated into up‐ and downregulated genes in the KOA group compared to the NO.KOA group. The colored bars display the ‘set size’ corresponding to the number of genes up‐ or downregulated within each cell type. The black horizontal bars display the intersection size corresponding to the number of overlaps of differentially expressed genes between cell types. Cell types with overlapping DEGs are connected with black lines (B) Volcano plot displaying differentially expressed genes in adipocytes between KOA and NO.KOA group. Genes with a positive log fold change are increased in the KOA group. Dots marked in orange have a log2 fold change above +1 or −1 and an adjusted *p*‐values < 0.05. Dots marked in gray have a log2 fold change above +1 or −1. (C) Dot plot visualizing top 10 selected up‐ and downregulated pathways enriched in adipocytes from individuals with KOA compared to the NO.KOA group. Significant pathways in the “Biological process” ontology gene set were determined by an overrepresentation test. The dot size corresponds to the number of genes involved in the specific pathway. The color of the dots corresponds to the FDR value, which is also displayed on the *x*‐axis.

We next examined DEGs between the two groups within each cell type cluster (Supporting Information File S1). In adipocytes, 367 genes were differentially expressed (FDR < 0.05, |log2FC| > 0.5) between the two groups with an upregulation of Serum Amyloid A1 (SAA1) and SAA2 in the KOA group (Figure 2A,B), both of which are secreted by adipocytes and are linked to obesity, inflammation, and metabolic and cardiovascular disease (Yang et al. 2006). Transcriptional differences between KOA and NO.KOA were unique for each cell type with minimal overlap of DEGs between cell types. Interestingly, a pathway analysis revealed significant enrichment of the biological processes related to lipid and fatty acid metabolism in adipocytes from the KOA group (Figure 2C). Downregulated pathways in the adipocyte population in the KOA group were primarily related to regulation of signaling and phosphorylation, as well as vascular and circulatory system development. In the progenitor, macrophage, and immune cell populations, few genes were differentially expressed between KOA and NO.KOA groups.

### 2.2 Associations Between Clinical Phenotypes and Cell Type Proportions

**FIGURE 3:**
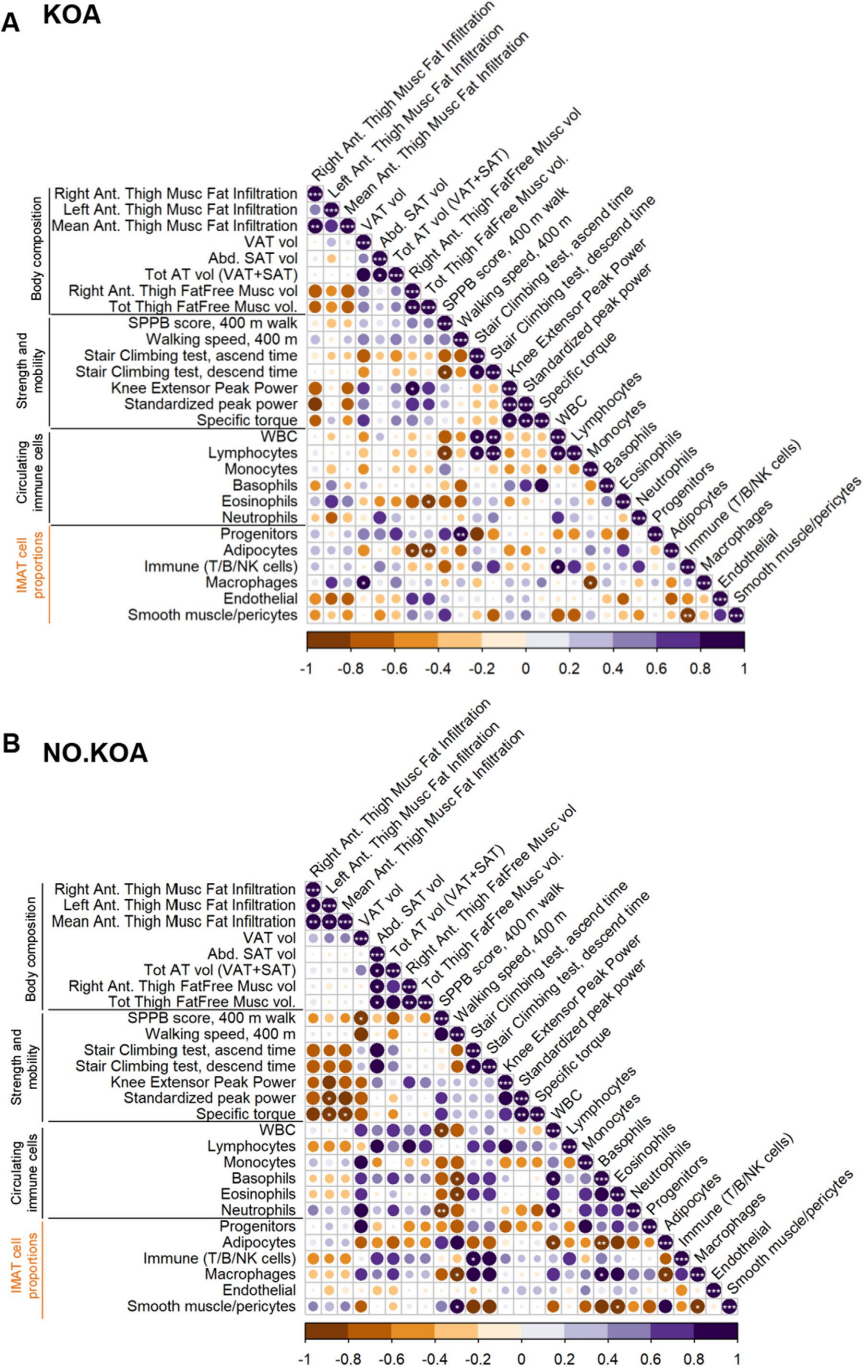
Correlation of clinical phenotype with cell type proportions stratified by KOA status. Correlation matrix of snRNA‐seq based cell type proportions (marked in orange) and relevant measures of body composition, muscle strength and mobility and absolute values of circulating immune cells in individuals with KOA (A) and without KOA (B). Pearson correlation coefficient was used to test the significance of the correlations. Pearson's R value (from −1 to 1) for each correlation is visualized by a color scale in which a purple color indicates a positive correlation and orange/brown color indicates negative correlation. *p* values: * < 0.05, ** < 0.01, *** < 0.001. Abd; abdominal, Ant; anterior, AT; adipose tissue, Musc, muscle; Tot, total; WBC, white blood cells; vol, volume; SAT, subcutaneous adipose tissue; VAT, visceral adipose tissue.

We next investigated the associations between the cell type proportions identified by snRNA‐seq and relevant measures of body composition, muscle strength and mobility, and circulating levels of immune cells stratified by KOA status (Figure 3A,B). In the KOA group, the proportion of adipocytes had a significantly negative correlation with right and total thigh fat free muscle volume (*p* < 0.05 and *p* < 0.01), whereas macrophages had a positive correlation with VAT volume (*p* < 0.05). The IMAT‐resident immune cell population displayed a significant correlation with absolute numbers of circulating white blood cells (WBC) (*p* < 0.05), suggestive of conformity between our snRNA‐seq data and the more systemic measure of immune cells and identifying IMAT as a potential source of these circulating immune cells. However, we cannot exclude the opposite scenario in which elevated levels of circulatory immune cells lead to greater infiltration of immune cells in peripheral tissues such as IMAT. Moreover, stair climbing descend time was positively associated with circulating WBCs and lymphocytes (*p* = 0.05–0.001), indicative of a slowed mobility with elevated levels of circulating immune cells. Interestingly, there was an inverse correlation between IMAT immune cells and smooth muscle/pericytes (*p* < 0.01) (Figure 3A). In the NO.KOA group, a few associations between IMAT cell type proportions were observed including a negative correlation between macrophages and adipocytes, as well as smooth muscle/pericytes (*p* < 0.05). IMAT‐resident macrophages moreover had a negative correlation with walking speed, which contrasted with the positive correlation observed with the proportion of smooth muscle/pericyte. Lastly, adipocytes had an inverse correlation with circulating WBC and basophils (*p* < 0.05 and *p* < 0.01). Although few strength and mobility traits correlated with IMAT cell type proportions, we did see an inverse correlation between leg power and IMAT abundance (*p* < 0.05), aligning with our previous finding in SOMMA (Distefano et al. 2024) (Figure 3B).

### 2.3 Sub‐Clustering of Immune and Progenitor Cell Populations

#### 2.3.1 Immune Cells

**FIGURE 4:**
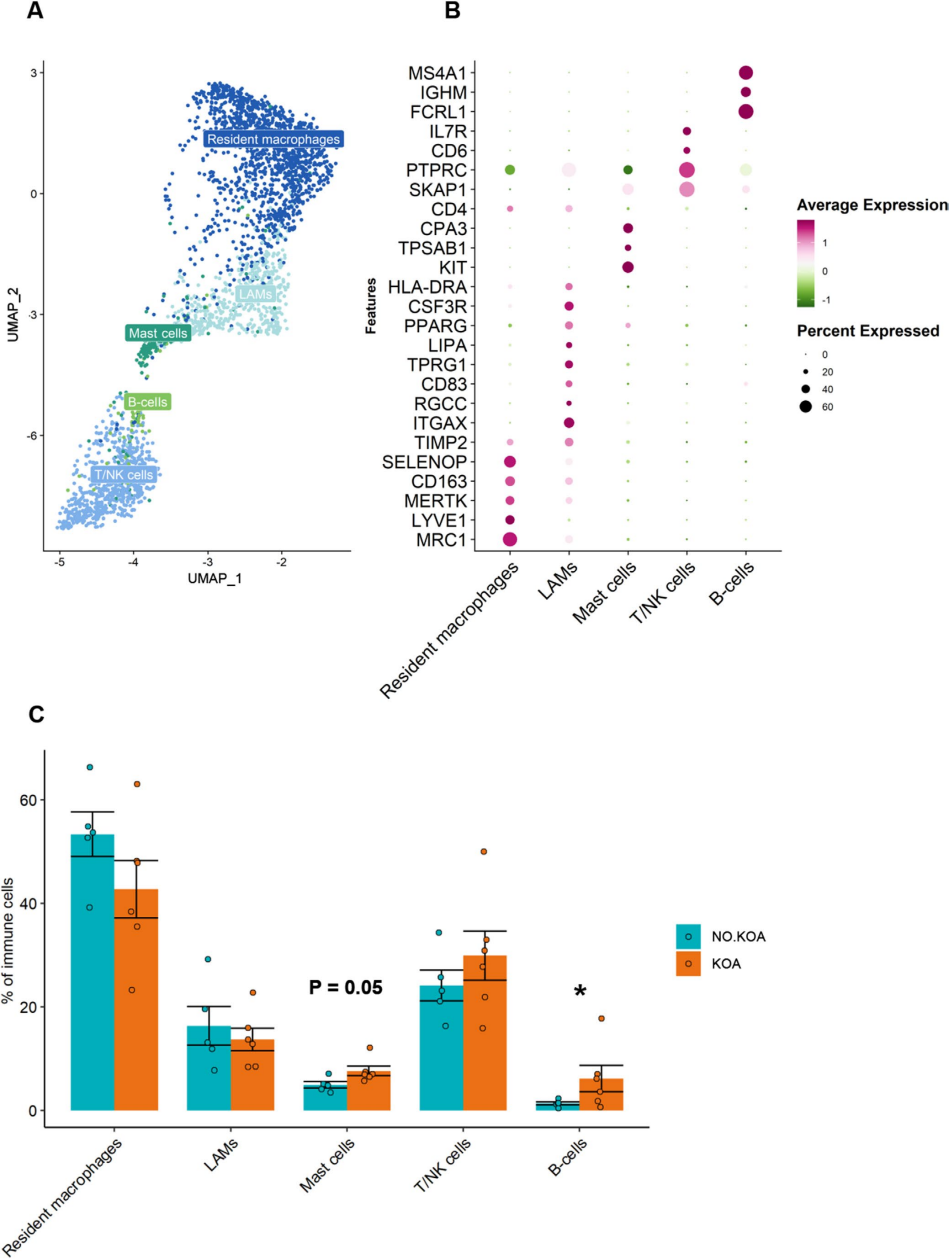
Sub‐clustering of immune cell populations. (A) UMAP displaying the sub‐clustering of the initial macrophage and immune cell populations giving rise to five sub‐clusters. (B) Dot plot showing the expression level of common gene markers for each of the five cell type clusters. The size of the dots corresponds to the percentage of cells in the cluster which express the specific gene marker. The color of the dot represents the average expression of the gene marker relative to the expression in the other clusters. (C) Bar plot showing the percentage of each of the cell types within the KOA (orange) and NO.KOA group (turquoise). *p*‐values reflects significance from a Welch's *t*‐test. *p* < 0.05 = *.

Performing a sub‐clustering of the immune (T/B/NK) and macrophage populations, we resolved two types of macrophages; Resident macrophages marked by expression of *MRC1*, *LYVE1*, *MERTK* and *CD163*, as well as lipid‐associated macrophages (LAMs) displaying markers related to lipid metabolism (*ITGAX*, *LIPA*, *PPARG*). Moreover, the immune cluster separated into T/NK cells (*SKAP1*, *PTPRC*, *IL7R*) and B cells (*IGHM*, *FCRL1*, *MS4A1*). Lastly, a subcluster of mast cells expressing *KIT*, *TPSAB1*, and *CPA3* was also identified (Figure 4A,B). Importantly, all individuals were represented in each immune population sub‐cluster. We found an increased abundance of B‐cells and mast cells in the KOA group (*p* = 0.04, *p* = 0.05, respectively), but none of the other immune cell subpopulations significantly varied by KOA status (Figure 4C).

#### 2.3.2 Progenitor Cells

**FIGURE 5:**
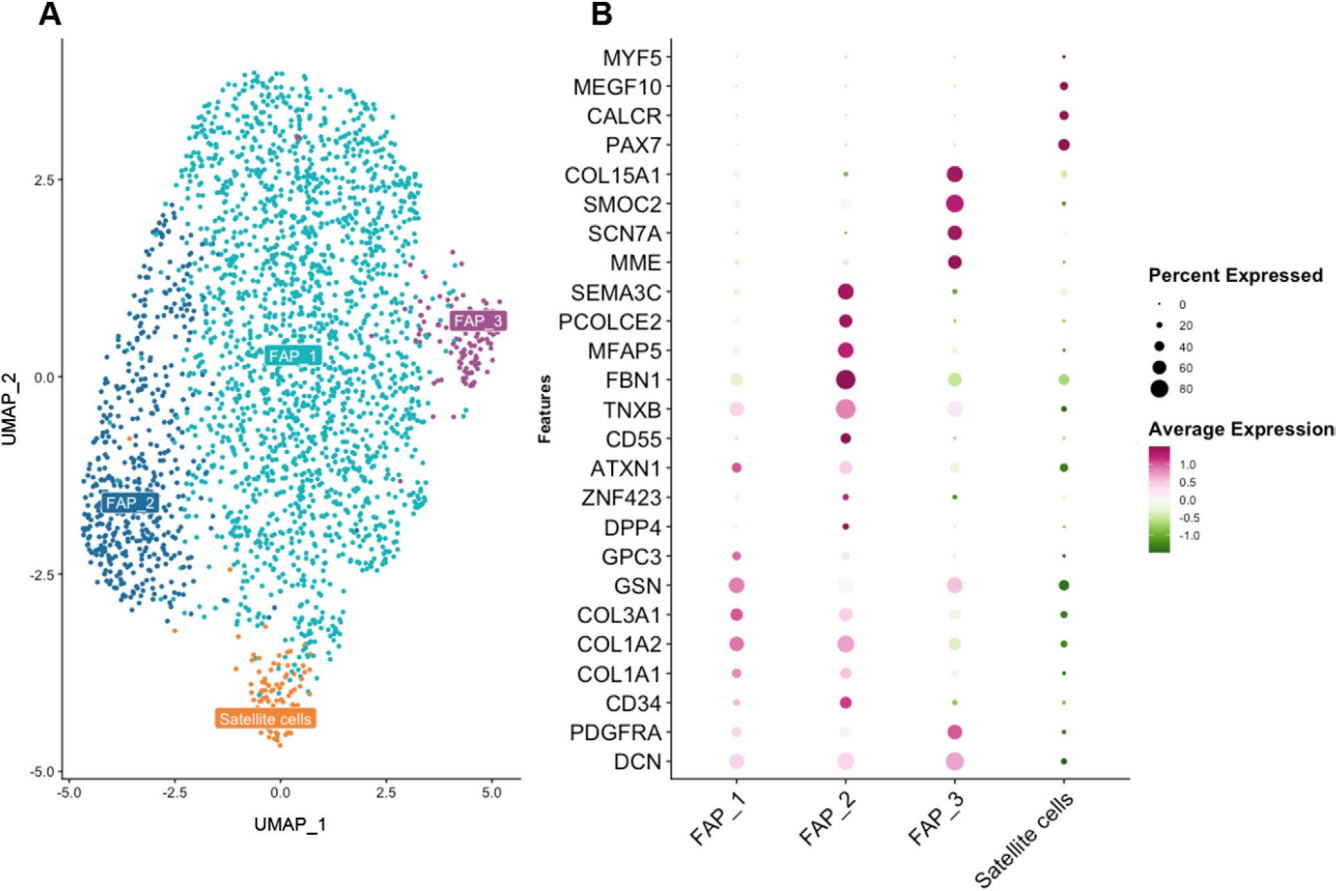
Progenitor cell sub‐clustering. (A) UMAP displaying sub‐clustering of the progenitor cell population, giving rise to four sub‐clusters. (B) Dot plot showing the expression of common gene markers for each of the four identified sub‐clusters. The size of the dots corresponds to the percentage of cells in the cluster which express the specific gene marker. The color of the dot represents the average expression of the gene marker relative to the expression in the other cell type clusters.

Sub‐clustering of the progenitor cell population, which has markers of stem cells and fibro‐adipogenic progenitors (FAPs), revealed three subpopulations of FAPs; FAP_1 (*GSN*, *COL1A2, COL3A1*), FAP_2 (*FBN1*), and FAP_3 (*MME*), as well as a small population of satellite cells (*PAX7*, *CALCR*, *MEGF10*) (Figure 5A,B). Notably, the markers of the FAP subtypes are in agreement with recently published single‐cell papers of skeletal muscle (De Micheli et al. 2020; Farup et al. 2021; Rubenstein et al. 2020) and aligned with FAP populations found in fatty infiltrated muscle of individuals with hip OA (Fitzgerald et al. 2023) (Figure S3). We did not find any compositional differences in the progenitor subpopulations between KOA and NO.KOA and few genes showed a robust differential expression between the groups (Figure S4).

### 2.4 Cell–Cell Interactions

**FIGURE 6:**
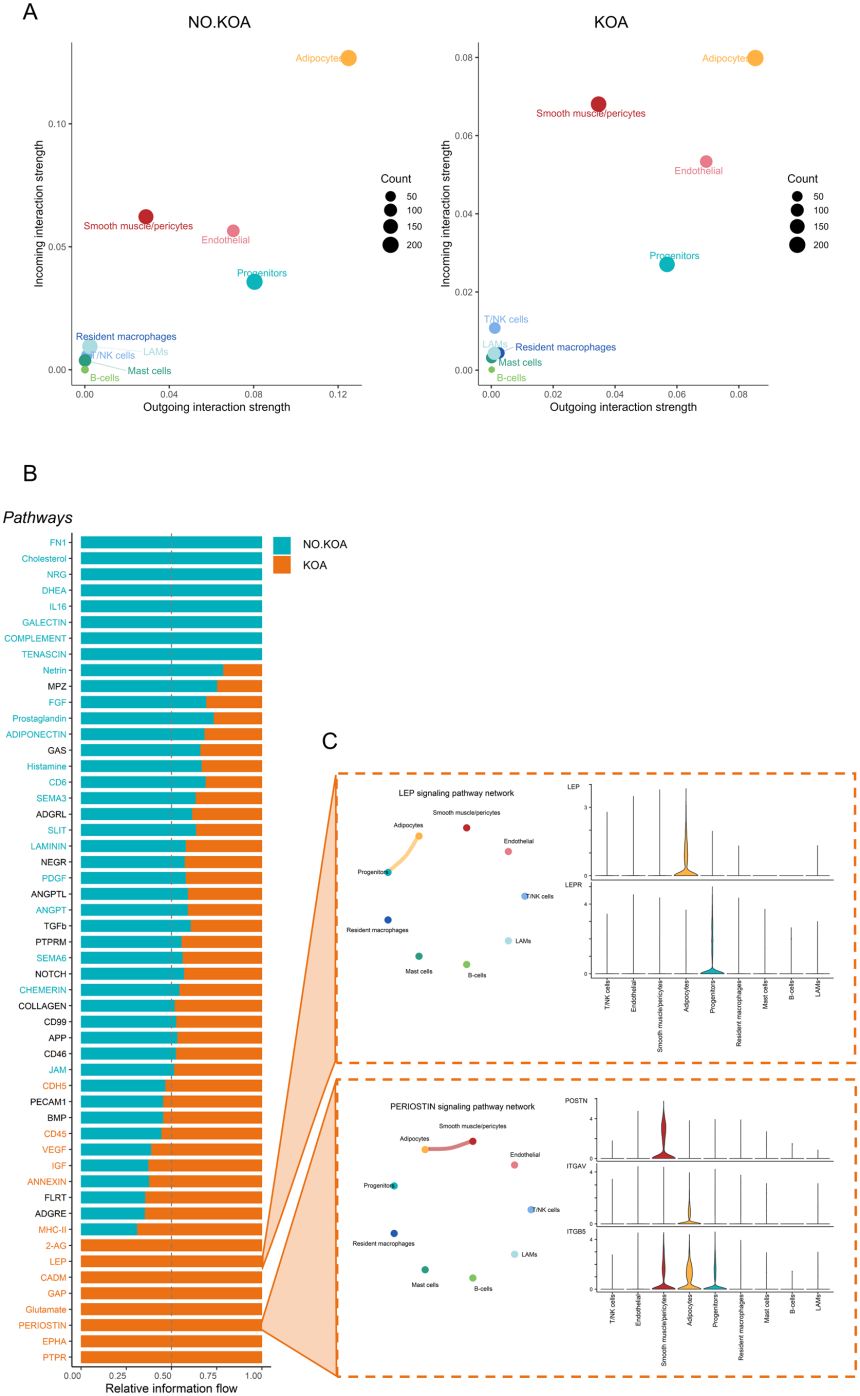
Cell–cell communication networks and signaling. (A) Scatter plots showing the signaling role of each cell type (outgoing‐ and incoming interaction strength) separated by KOA and NO.KOA group. The *x*‐ and *y*‐axis show the total outgoing and incoming communication probability associated with each cell type. The dot size is proportional to the number of outgoing and incoming signals associated with each cell type (B) Bar chart displaying the significant signaling pathways ranked based on the difference in the overall information flow (i.e., interaction strength of each signaling pathway) between KOA and NO.KOA. The top signaling pathways colored in turquoise are enriched in the NO.KOA group. The lower signaling pathways colored in orange are enriched in the KOA group. (C) Circle and violin plots of LEP and POSTN signaling pathways enriched in the KOA group. The color of the line in the circle plot specifies which cell type the signal is coming from. The thickness of the line corresponds to the interaction strength. Thicker edge line indicates stronger signal.

Lastly, we used CellChat (Jin et al. 2021) to infer cell–cell communication networks between the cell types in IMAT based on estimated over‐expressed ligand‐receptor interactions. Examining the signaling role of each cell type across all detected signaling pathways, we found that the adipocyte population was by far the cell type with the greatest intercellular communication probability of outgoing and incoming signals, independent of KOA status (Figure 6A). In general, the immune populations had a minor contribution to the overall signaling network in both groups.

We found a diverse set of putative ligand‐receptor interactions enriched in both groups (Figure 6B). In the KOA group, Protein Tyrosine Phosphatase Receptor (PTPR), Ephrin‐A (EPHA), Periostin (POSTN), Glutamate, Gap junction, Cell Adhesion Molecule 1 (CADM1), Leptin (LEP) and 2‐Arachidonoylglycerol (2‐AG) signaling were significantly and exclusively enriched (Figure 6B). Signaling of the proinflammatory adipokine LEP derived specifically from the adipocytes and communicated only with the progenitor cell population via the leptin receptor (LEPR) (Figure 6C), which displayed an enhanced expression compared to the NO.KOA group (Figure S6). POSTN signaling was driven by the smooth muscle/pericyte population to ITGAV and ITGB5 receptors on the adipocytes (Figure 6C). Several of the remaining hits in the signaling pathways exclusive to the KOA group (PTPR, CADM1, Glutamate) displayed a large degree of autocrine signaling in adipocytes (Figure S4). The expression level of receptors and their ligands in the two groups for each of the KOA enriched pathways can be found in Supporting Information, Figure S6.

Fibronectin 1 (FN1), Cholesterol, Neuregulin (NRG), Dehydroepiandrosterone (DHEA), interleukin 16 (IL‐16), Galectin, Complement and Tenascin signaling were exclusively and significantly enriched in the NO.KOA group (Figure 6B). Interestingly, the outgoing signals of these pathways derived mainly from LAMs and progenitor cells, except for IL‐16 and NRG, which involved the adipocyte population (Figure S5). The expression level of receptors and their ligands in the two groups for each of the NO.KOA enriched pathways are presented in Supporting Information, Figure S8.

## 3 Discussion

We interrogated the cell composition and cell‐specific transcriptional profiles within IMAT of a cohort of older women from the SOMMA KOA ancillary study with and without KOA and knee pain. We identified six major cell types in IMAT, with subpopulations including mast cells, B‐cells, resident macrophages, LAMs, satellite cells and three FAP populations. We found a greater abundance of mast and B‐cells in IMAT from the KOA group. Differential gene expression analysis identified the adipocyte population as the most transcriptionally distinct cell type between KOA and NO.KOA groups. Exploring cell–cell communication networks, we discovered that adipocytes had the most prominent signaling role of all identified cell types and that pro‐inflammatory signaling was enriched in individuals with KOA.

The cellular composition of human IMAT is unknown compared to other AT depots. Studies using similar nuclei isolation methodology have reported a lower adipocyte proportion in VAT (~15%–20%) compared to ASAT (~25%–30%) (Emont et al. 2022; Strieder‐Barboza et al. 2022), whereas we find the adipocyte proportion in IMAT to be intermediate (~22%). A large proportion of the IMAT cells belonged to the vasculature, with ~25% endothelial cells and ~18% smooth muscle cells and pericytes. A similar proportion of endothelial cells has been found in ASAT, whereas a substantially lower proportion is found in VAT (4%–7%) (Emont et al. 2022; Strieder‐Barboza et al. 2022). Smooth muscle cells and pericyte populations have also been reported in other single‐nuclei studies of AT depots, however, at much lower proportions than what we identified in IMAT (Emont et al. 2022; Massier et al. 2023). Pericytes not only orchestrate vascular development, but are also adipogenic precursors (Picoli et al. 2024; Tang et al. 2008; Tran et al. 2012), while smooth muscle cells have been reported as a potential origin of beige adipocytes (Long et al. 2014). Our IMAT‐resident progenitor cell population had a lower proportion than what has been reported in ASAT (15% vs. 18%–37%) (Emont et al. 2022; Massier et al. 2023; Strieder‐Barboza et al. 2022; Whytock et al. 2022, 2024). However, we identified three FAP subpopulations of which one displayed identical gene markers as a FAP subpopulation in a snRNA‐seq study of fatty infiltration in muscle from individuals with hip OA (Fitzgerald et al. 2023). This FAP population was suggested as a potential origin of the adipocytes present in IMAT due to its high adipogenic potential upon in vitro differentiation. Taken together, these findings indicate that IMAT may harbor a larger and more diverse reservoir of adipogenic progenitor cells, such as pericytes and smooth muscle cells, than other AT depots that have larger pools of preadipocytes. As such, the vasculature in IMAT could serve as an additional reservoir and niche for progenitor cells.

In the context of KOA, VAT and ASAT have only been studied in terms of quantity as it relates to disease development, progression and burden (Li, Schwartz, et al. 2020), leaving a gap in understanding the molecular quality of AT depots in this condition. Recent studies have described the cellular heterogeneity of the infrapatellar fat pad (IFP), located directly at the knee joint. Adipocytes comprised ~16%–23% of the total cells in the IFP and were classified into three to four subtypes, resembling those found in other white adipose depots (Peters et al. 2024; Pu et al. 2024; Tang et al. 2024). Resident macrophages and LAMs identified in IMAT from our cohort of older women displayed similar gene markers as those found in the IFP and synovium tissue (Peters et al. 2024; Tang et al. 2024). We found no proportional differences in the macrophage subtypes between the KOA and NO.KOA groups, however, a higher percentage of inflammatory macrophages has been reported in the IFP of KOA individuals (Tang et al. 2024). Moreover, we previously found that older adults, with a similar age to the women included in this study, have a higher abundance of macrophages, in particular LAMs, in ASAT, compared to younger adults (Whytock et al. 2024). Hence, we speculate that macrophage subpopulations within the adipose depots around the knee joint, such as IFP and IMAT, may contribute to local inflammation at the knee joint and may therefore play a key role in the development and progression of KOA.

The immune cell composition of IMAT in our study aligns with that observed in ASAT and VAT from other snRNA‐seq studies (Emont et al. 2022; Strieder‐Barboza et al. 2022; Whytock et al. 2024). We found an increased abundance of mast cells and B‐cells in IMAT from KOA individuals. Mast cells are recognized as a key component in the innate immune system contributing to the chronic low‐grade inflammation implicated in OA (Robinson et al. 2016). An increased mast cell density in OA synovium was observed over 20 years ago (Buckley et al. 1998; Dean et al. 1993; Pu et al. 1998), yet only recently have synovial mast cells in KOA been characterized and stratified into pathotypes using scRNA‐seq (Zhao et al. 2022). Several single‐cell studies have reported mast cells in the IFP of individuals with KOA (Pu et al. 2024; Tang et al. 2024), and the mast cell percentage has been shown to be higher in the IFP compared to the ASAT depot (Klein‐Wieringa et al. 2011). B‐cells have likewise been identified in the synovium and IFP of individuals with KOA but represent only a small fraction of the infiltrating cells (Burt and Scanzello 2023; Pu et al. 2024; Tang et al. 2024). One of the most important findings of our current study is not only the presence of mast cells and B‐cells in human IMAT and their greater abundance in KOA, but in our cell–cell communication analysis, we only found minor signaling output from mast and B‐cells to other IMAT cell types. We speculate that IMAT might serve as a reservoir for these specific immune cells for both plasma and the synovium, where they can play an active role.

Adipocytes were the most transcriptionally distinct cell type between the KOA and NO.KOA groups. Among the top upregulated genes in adipocytes from the KOA group were SAA1 and SAA2. We and others have identified an adipocyte subtype that expresses greater SAA1 and SAA2 and displays a pro‐inflammatory phenotype (Bäckdahl et al. 2021; Whytock et al. 2024), which aligns with the more inflammatory phenotype of IMAT, we observed in our women with KOA. Our pathway analysis showed an upregulation of lipid and fatty acid metabolism in adipocytes from the KOA group. These transcriptional features characterize an adipocyte equipped for lipid storage and turnover, which in ASAT is considered a ‘healthy’ phenotype (Hammarstedt et al. 2018; Sakers et al. 2022), but could be indicative of an AT depot poised for expansion and thus detrimental within skeletal muscle tissue as previously shown in obesity and metabolic disease (Goodpaster et al. 2023). Interestingly, the downregulation of circulatory system and vasculature development pathways could indicate inhibited angiogenesis through decreased release of angiogenic factors from adipocytes, which can lead to a hypoxic environment and subsequent rises in inflammation (Trayhurn 2013).

Obesity is thought to increase the risk of KOA and knee pain through mechanical loading on the knee joint and release of inflammatory mediators from dysfunctional AT (Binvignat et al. 2024). A recent study showed that a 26‐week GLP‐1 receptor agonist treatment in obese individuals with KOA and knee pain significantly reduced knee pain, which could only partly be explained by the coinciding weight loss (Henning et al. 2024). Additionally, epidemiologic studies have reported that in individuals with KOA, abdominal AT depots, primarily visceral, are associated with knee pain (Li, Schwartz, et al. 2020). However, inflammatory mediators arising from other adipose depots could similarly affect the knee joint. Here, secretion of pro‐inflammatory mediators from IMAT of the thigh, which is located much closer to the knee joint, is a plausible candidate contributing to activation of nociceptors and knee pain. Several studies have reported direct activation and sensitization of nociceptor neurons by TNF‐α, IL‐1β, and IL‐6 (Binshtok et al. 2008; Ebersberger and Schaible 2025; Schaible 2014) and synovial fluid levels of these cytokines has been shown to correlate with pain scores, particularly in the early stages of KOA (Li, Li, et al. 2020). Given that IMAT exhibits a more pro‐inflammatory profile compared to other adipose depots (Kahn et al. 2022), it is plausible that cytokines derived from this depot contributes to knee pain in KOA. This is underlined by the fact that the mean BMI of the women in our study was 24, demonstrating that it is not only mechanical loading and stress from excess adiposity that contributes to their knee pain. Hence, in the older women with KOA studied herein, pro‐inflammatory signaling might partly contribute to knee pain.

We found an enrichment of leptin signaling in the KOA group, driven by an enhanced expression of LEPR in the progenitor cell population compared to the expression levels in the NO.KOA group (Figure S6). Leptin is released from adipocytes and plays a major role in energy homeostasis, as well as in immune and inflammatory processes (Palhinha et al. 2019). Our analysis suggested that adipocytes in the KOA group mainly relayed information to the progenitor cell population. We speculate that this interaction promotes adipogenesis in the underlying FAP populations, as LEPR signaling can promote adipogenesis of skeletal stem cells (Yue et al. 2016). Serum leptin levels are positively associated with IMAT mass (Vella and Allison 2018), and numerous studies have reported increased leptin levels in individuals with KOA and knee pain (Askari et al. 2020; Bas et al. 2014; Kroon et al. 2019; Min et al. 2021). Moreover, dysfunctional leptin signaling has been shown to modulate the nociceptive response in acute inflammatory pain, underlining its role in pain sensation (Hu et al. 2018). Finally, in a recent review, leptin was identified as a BMI‐independent factor contributing to OA‐related pain (Binvignat et al. 2024), with the largest effect seen in women (Kroon et al. 2019). Given that we find increased expression of leptin signaling in the IMAT, this could be a local effect on the muscle tissue and nearby knee joint, as opposed to a more systemic effect.

To our knowledge, no studies to date have explicitly examined the cell‐type composition of IMAT in relation to pain status in KOA. While our study lacks a KOA group without pain, the observations in the pain group allow cautious speculation about potential pain‐related differences in the cellular phenotype. We speculate that pro‐inflammatory cells are more abundant in individuals with KOA and pain, contributing to cytokine and immune‐mediated sensitization of nociceptors. Additionally, the abundance of different FAP subtypes could affect muscle tissue strength through promoting extracellular matrix deposition or IMAT expansion, thereby affecting knee joint functionality and potentially causing knee pain. Future research will need to validate these speculations.

### 3.1 Strength and Limitations

We obtained IMAT from a deeply phenotyped cohort of older women from the SOMMA KOA ancillary study, with good racial representation. The methods for collecting and storing the biopsies were outstanding, resulting in high‐quality specimens. The use of snRNA‐seq is cutting edge leading to novel findings in human IMAT. However, correlation analyses between cell type proportions should be interpreted with caution, as relative increases in one population inherently reduce the proportions of others. Moreover, the small sample size of only older women limits generalizability to men and younger women; though, women are affected by KOA to a greater degree. These observations are cross‐sectional, representing a single point in time, in a dynamic disease course. Given that SOMMA is an ongoing longitudinal study, we will expand our sample to include both men and women across the lifespan in future studies. Lastly, the absence of intermediate groups (i.e., KOA without pain and NO.KOA with pain) limits our ability to separate the effects of disease state and pain and should be a topic for future research.

### 3.2 Conclusion

This is the first report of the cellular heterogeneity of human IMAT in individuals with KOA. Our results highlight the adipocytes as the most transcriptionally diverse cell type in IMAT between women with and without KOA and as a major signaling source, with the potential to impact the surrounding muscle tissue and joint. We observed notable similarities in the cellular composition, transcriptional profile, and inferred secretory environment of IMAT and the environment reported in the knee joint of individuals with KOA, highlighting IMAT as a potential source of inflammation in KOA.

## 4 Methods

### 4.1 Study Population

This study is an ancillary study to the Study of Muscle, Mobility and Aging (SOMMA) which was designed to examine the biological processes contributing to changes in mobility and fitness with aging (Cummings et al. 2023). For this ancillary study, SOMMA participants who returned for the one‐year follow‐up visit and consented for a standing knee radiograph of both knees were enrolled. For snRNA‐seq analysis, a subgroup of women was selected based on having a Kellgren‐Lawrence score between 0 and 1 (NO.KOA) or 3‐4 (KOA) and a muscle biopsy enriched for IMAT (Figure S6). All participants provided written informed consent, and the study was approved by the Western Institutional Review Board (WCGIRB #20180764) for all participating sites (Cummings et al. 2023). Clinical measures presented in the paper were performed as described in Cummings et al. (Cummings et al. 2023) and briefly outlined below. Data as of November 2023 were included in the analyses described in the current paper.

### 4.2 Blood Collection and Complete Blood Count Measures

Blood samples were drawn after an overnight fast from an antecubital vein using a blood collection set (BD, 367326). Complete Blood Count measures were performed at Local Quest Diagnostic Laboratory in Pittsburg or Wake Forest Medical Center laboratory and included platelet count, red blood cell count, white blood cell count, hemoglobin density, mean corpuscular hemoglobin (MCH), mean corpuscular hemoglobin concentration (MCHC), mean corpuscular volume (MCV), hematocrit, red cell distribution width, and 5 different kinds of white blood cells.

### 4.3 MRI Imaging

A whole‐body MRI scan was taken to assess body composition including quadriceps muscle volume, muscle fat infiltration (% fat by proton density fat fraction), total thigh muscle volume, fatty liver, distribution of visceral and subcutaneous adipose tissue. MR data was acquired with the Dixon water‐fat imaging method. MRI images were analyzed using AMRA Researcher (AMRA Medical AB, Linköping Sweden) and consisted of image calibration (Romu et al. 2011), fusion of image stacks, image segmentation (Karlsson et al. 2015), and quantification of fat and muscle volumes (Borga et al. 2020; Linge et al. 2019; West et al. 2018) as well as manual quality control by a blinded trained operator.

### 4.4 D3‐Creatine Muscle Mass Determination

Whole‐body D3‐creatine (D3‐Cr) muscle mass was measured in participants using a D3‐creatine dilution protocol. Briefly, participants ingested a tablet with 30 mg of D3‐Cr and provide a fasting, morning urine sample 72–144 h later (Clark et al. 2014; Shankaran et al. 2018). In the urine sample, D3‐Cr, unlabeled creatinine, and creatine were measured using high‐performance liquid chromatography (HPLC) and tandem mass spectroscopy (MS/MS). These measures were used to determine total body creatine pool size and thereby skeletal muscle mass.

### 4.5 Muscle Strength and Mobility Tests

An expanded version of the Short Physical Performance Battery (SPPB) was used (Guralnik et al. 2025), including a timed 4 m walk, chair stands, balance test, and short narrow walk. Leg extension power and strength (one repetition maximum, 1RM) were assessed using the Keiser AIR300 or A420 Leg Press system. A timed stair climbing test was performed, where participants were asked to climb up and down 4 stairs, 3 consecutive times to measure functional leg power (Lange‐Maia et al. 2019). Walking speed was measured by timing a 400 m walk, where participants were asked to walk at their usual pace. Inability to walk 400 m within 15 min without assistance, was considered a major mobility disability (Fielding et al. 2011). Steps per day were measured using activPAL4 micro (PAL Technologies Ltd., Glasgow, Scotland).

### 4.6 IMAT Biopsies

A fasting percutaneous biopsy from the vastus lateralis muscle was obtained using a Bergström needle (5 or 6 mm) with suction and under local anesthetic (1 or 2% lidocaine HCL) as previously described (Zamora et al. 2024). IMAT was dissected free from muscle and connective tissue and snap‐frozen in liquid nitrogen before storage at −80°C until use.

### 4.7 Nuclei Isolation From Intermuscular Adipose Tissue

Nuclei were isolated from frozen IMAT as described in detail previously (Elingaard‐Larsen et al. 2024). In brief, IMAT (50 mg) was pulverized under liquid nitrogen before being homogenized with a glasscol homogenizer in 1 mL of homogenization buffer (5 mM MgCl2, 10 mM Tris Buffer pH 8.0, 25 mM KCL, 250 mM sucrose, 1 μM DDT, 1 × protease inhibitor, 0.4 U/μL SUPERase·In RNase Inhibitor (Thermofisher Scientific) in nuclease‐free water). Triton‐X100 (0.1% v/v) was added to the homogenate and left to incubate on ice for 10 min with regular vortexing. Samples were then firstly filtered through a 100 μm strainer followed by a 40 μm strainer (BD Falcon). Next, samples were centrifuged at 2700*g* for 10 min at 4°C, re‐suspended in 500 nuclei isolation medium (0.1 mM EDTA, 0.4 U/μL Ribolock RNAase inhibitor, 1% BSA in PBS −/−) and centrifuged again at 1000*g* for 10 min at 4°C. The pellet was re‐suspended in 200 μL nuclei isolation medium before centrifugation at 1000*g* for 10 min at 4°C. The nuclei were stained with DAPI (ReadyProbes Cell Viability Imaging Kit, Thermofisher Scientific) and filtered through a 30 μm strainer (MACS Smartstrainer, Miltenyi Biotec) before being counted with a countess II automated cell counter (Thermofisher Scientific).

### 4.8 Library Preparation and Sequencing

Single nuclei suspension, prepared for a targeted nuclei recovery of 10,000 nuclei, was loaded to a Chromium Chip G (10X Genomics) for generation of Gelbeads‐in‐Emulsion (GEMs) followed by library preparation performed using the 10X Chromium Next GEM Single Cell 3′ Reagent Kit v3.1 (Dual index) (CG000315 Rev. E) according to the manufacture's description. The quality and concentration of the resulting cDNA libraries were assessed by electrophoresis (Agilent Bioanalyzer high sensitivity DNA chips). The libraries were sequenced on the NovaSeq X Plus platform using run cycle 151 + 10 + 10 + 151 and targeting 150G raw data per sample.

### 4.9 Bioinformatic Analyses

The bioinformatics approach applied in this manuscript largely follows the steps described in Whytock et al. (Whytock et al. 2024). Sequencing reads were mapped, aligned, and counted using CellRanger Analysis Pipeline (10× Genomics, V6.1.1) with GRCh38 as the reference genome. Initially, each individual sample underwent quality control (QC) in which the following parameters were used for filtering out low quality nuclei; < 200 genes, > 10,000 genes, > 10% mitochondrial reads and a cell complexity score < 0.80 (log10(number of genes)/log10(number of counts)). SCTransform from the *Seurat* R package was used for data normalization (Hafemeister and Satija 2019), followed by initial clustering of each sample using 2000 highly variable genes to detect sample dependent cell type clusters. Next, doublets were identified and removed using *DoubletFinder*. Lastly, data was adjusted for ambient RNA using *decontX* (Yang et al. 2020) for each sample separately. Nuclei with < 100 genes expressed after ambient RNA adjustment were removed from the dataset. Integration of the data from all 11 samples was performed using *harmony* (Korsunsky et al. 2019), which presented with the best adjusted rand index (ARI) (Hubert and Arabie 1985) and Average Local Inverse Simpson's index (LISI) (Korsunsky et al. 2019) score compared to other tested integration methods including STACAS and RPCA. Batch effect was furthermore assessed visually by plotting a UMAP color‐coded by study ID (Figure S7), to ensure no cell cluster derived from a single participant. We further assessed the quality of the integration, by comparing the cell type annotation before and after integration of the data (Figure S8). We found that a small population of nuclei shifted from being annotated as ‘myonuclei’ in the individual sample to “adipocyte” in the integrated dataset. These nuclei were removed from the dataset, as it is indicative of contamination/low quality nuclei.

To identify and validate cell type clusters, differential gene expression analysis between cell clusters was performed using a Wilcoxon rank sum test with *Seurat*'s “FindMarkers” function with an FDR cut‐off of < 0.05 and log_2_ FC > 0.25 or < −0.25. Differences in the proportion of each cell type between groups was determined using the linear framework integrated in the propeller function in the R package speckle (Phipson et al. 2022). Differential gene expression analysis between the KOA and NO.KOA group was performed using a Wilcoxon rank sum test with *Seurat*'s “FindMarkers” function with an FDR cutoff of < 0.05, log_2_ FC > 0.5 and only genes detected in minimum 10% of the nuclei in either of the two populations. Overrepresentation analysis of up‐ and downregulated genes (−0.5 > log_2_ FC > 0.5) in the KOA and NO.KOA group for each cell type was performed using a hypergeometric test from the R package *HypeR* (Federico and Monti 2020) using datasets from the Molecular Signatures Database (MSigDB) including Hallmark and Ontology gene sets and Reactome (Gillespie et al. 2022; Liberzon et al. 2015). Genes detected in each cluster was used as a background reference. Overrepresented pathways were considered significant at an FDR < 0.05.

### 4.10 CellChat Analysis

Cell–cell communication analysis was performed using *CellChat* version 1.6.1 (Jin et al. 2021). The average gene expression was calculated using the default method ‘trimean’ and overexpressed ligand and receptor genes and interactions were identified. A communication probability score for each interaction was calculated using the computeCommunProb function including the argument group.size = TRUE, to account for differences in proportion of the cell types. Cell–cell communication with < 10 cells in each cell group was filtered out. To compute communication probability on a signaling pathway level, the function computeCommunProbPathway was used. The function netAnalysis_signalingRole_scatter was used to identify and plot the signaling role of the different cell types in a scatter plot. To identify KOA and NO.KOA specific signaling pathways, the “rankNet” function was used to compare the signaling information flow for each signaling pathway, applying a paired Wilcoxon test to test for significance (*p* < 0.05). Circle plots created with the netVisual_aggregate function were used to visualize cell–cell communication of pathways of interest.

### 4.11 Statistical Testing

A Welch's *t*‐test was used for comparisons of clinical data between the KOA and NO.KOA group. *p* < 0.05 was considered significant. Pearson correlation coefficient was used to test the significance of correlations between IMAT cell type proportions and selected body composition measures, strength and mobility traits, and absolute numbers of circulating immune cells.

## Author Contributions

**Line O. Elingaard‐Larsen:** methodology, investigation, formal analysis, visualization, writing – original draft. **Katie L. Whytock:** methodology, investigation, formal analysis, visualization, writing – review and editing. **Adeline Divoux:** methodology, investigation, writing – review and editing. **Cheehoon Ahn:** writing – review and editing. **Giovanna Distefano:** writing – review and editing. **Bret H. Goodpaster:** writing – review and editing. **Paul M. Coen:** writing – review and editing. **Jamie N. Justice:** writing – review and editing. **Erin E. Kershaw:** writing – review and editing. **Nancy E. Lane:** conceptualization, methodology, supervision, funding acquisition, writing – review and editing. **Lauren M. Sparks:** conceptualization, methodology, supervision, funding acquisition, writing – review and editing.

## Funding

This work was supported by National Institute on Aging, R01AG059416, R01AG066474, R01AG070647, P30AG024827, P30AG021332. National Center for Advancing Translational Sciences, UL10TR001420.

## Conflicts of Interest

The authors declare no conflicts of interest.

## Supporting information


**Data S1:** acel70348‐sup‐0001‐DataS1.xlsx.

**Data S2:** acel70348‐sup‐0002‐FigureS1‐S13.pdf.

## Data Availability

snRNA‐seq data generated during this study will be made available at https://sommaonline.ucsf.edu/. Scripts used to process the snRNA‐seq data in this paper will be made available at GitHub (https://github.com/L‐OEL/IMAT_KOA).
