# Supplementary material for: Single‐Nuclei Resolution of Intermuscular Adipose Tissue Indicates an Inflammation‐Associated Cellular Profile in Individuals With Knee Osteoarthritis: Findings From the SOMMA KOA Ancillary Study
Source: Aging Cell. 2025 Dec 26;25(1):e70348. doi: 10.1111/acel.70348 (PMC12741905; doi:10.1111/acel.70348)
Supplement: Supplementary file 2 — Data S2: acel70348‐sup‐0002‐FigureS1‐S13.pdf. [file ACEL-25-e70348-s002.pdf]

Supplementary Figure S1

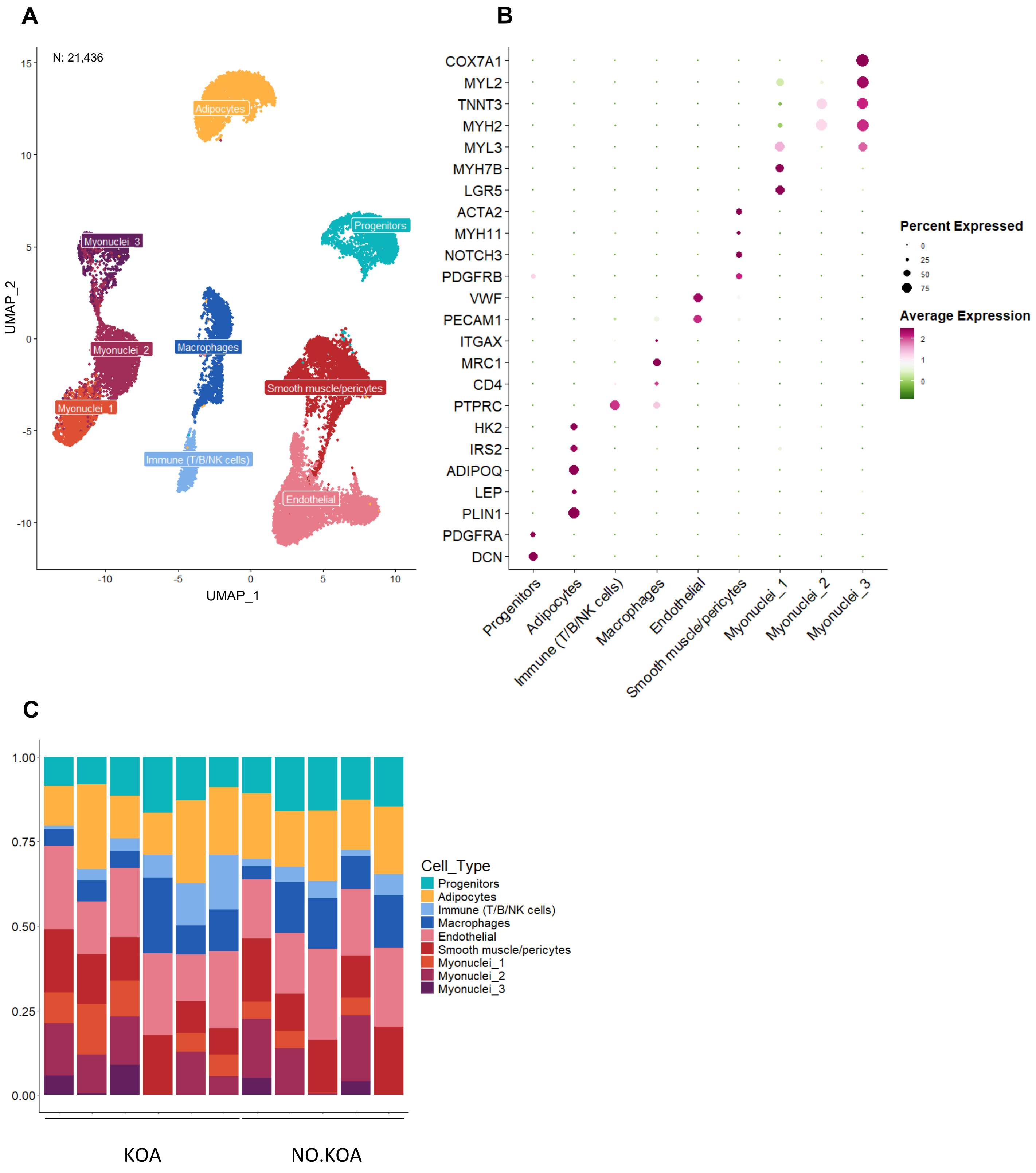

Figure S1: Identification of nine cell type clusters in IMAT **A)** UMAP depicting the nine cell type clusters identified based on 21,436 nuclei from in total 11 IMAT samples. Each dot represents a nucleus. N refers to the total number of nuclei used for clustering **B)** Dot plot displaying common gene markers for each of the nine cell type clusters. Dot size represent the percentage of cells in the cluster which express the specific gene marker, whereas the color of the dot represents the average expression of the gene marker relative to the other cell type clusters **C)** Stacked bar plot demonstrating the proportion of each the identified cell types for each study participant ordered according to group.

Supplementary Figure S2

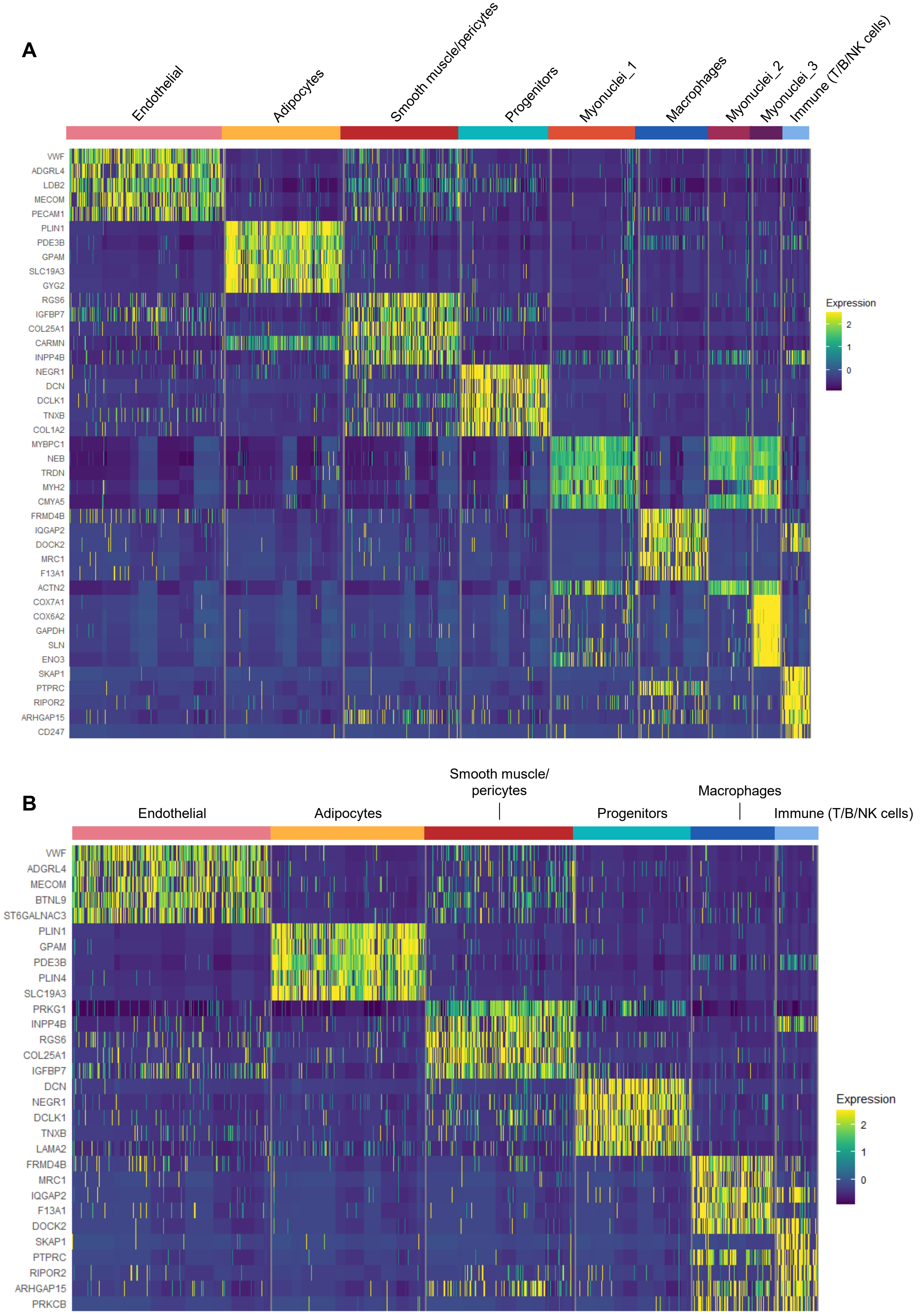

Figure S2: Heatmap of the top five differentially expressed genes for each identified cell type. **A)** The initial nine cell type clusters **B)** The final six cell type clusters excluding myonuclei clusters. Color indicates gene expression level.

Supplementary Figure S3

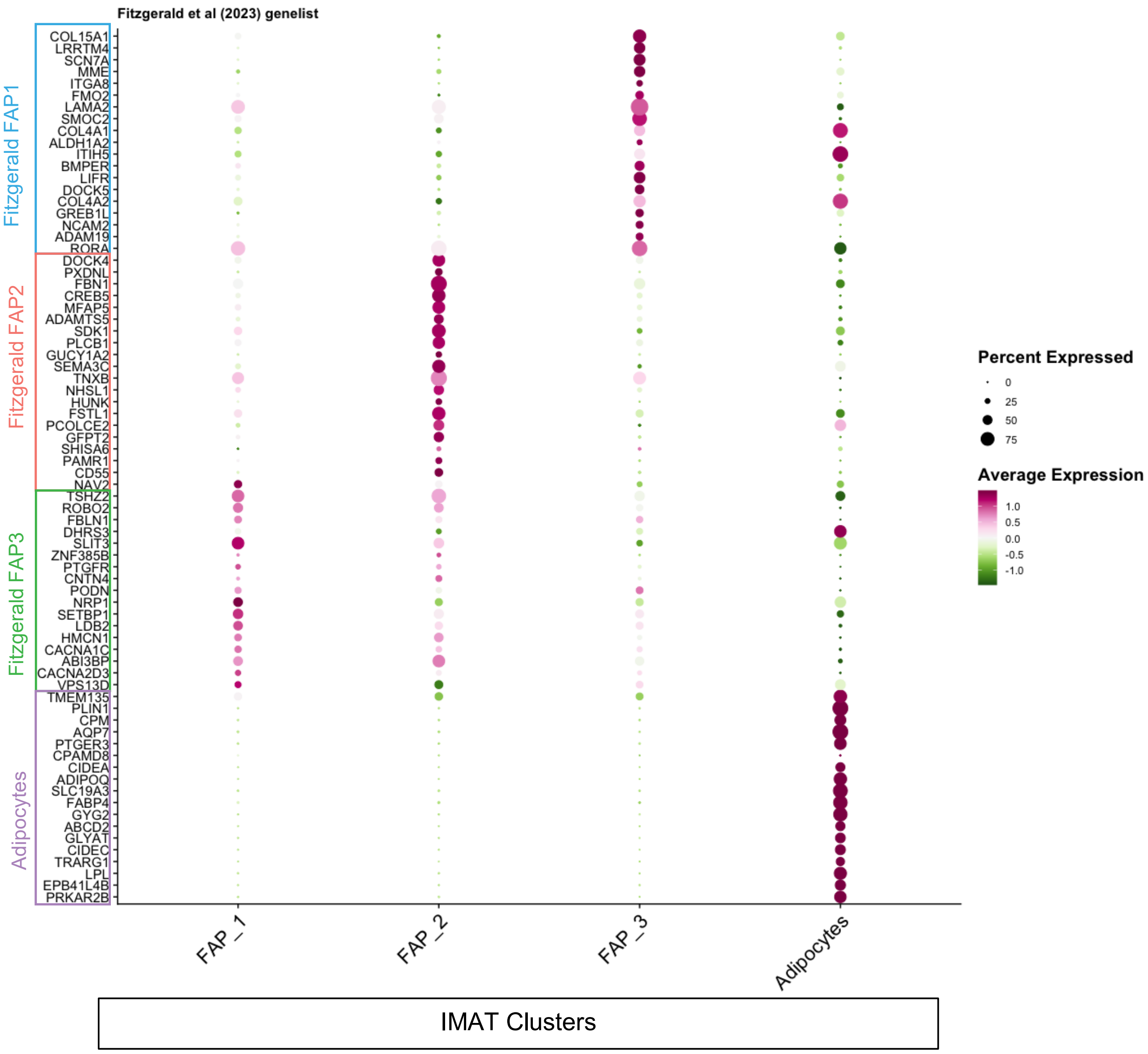

Figure S3: DotPlot of top enriched marker genes from Fitzgerald et al. 2023 in relation to average gene expression of fibro-adipogenic progenitors and adipocyte clusters from our dataset. Dot size represent the percentage of cells in the cluster which express the specific gene marker, whereas the color of the dot represents the average expression of the gene marker relative to the other cell type clusters

Supplementary Figure S4

FAP_1 - KOA vs NO.KOA

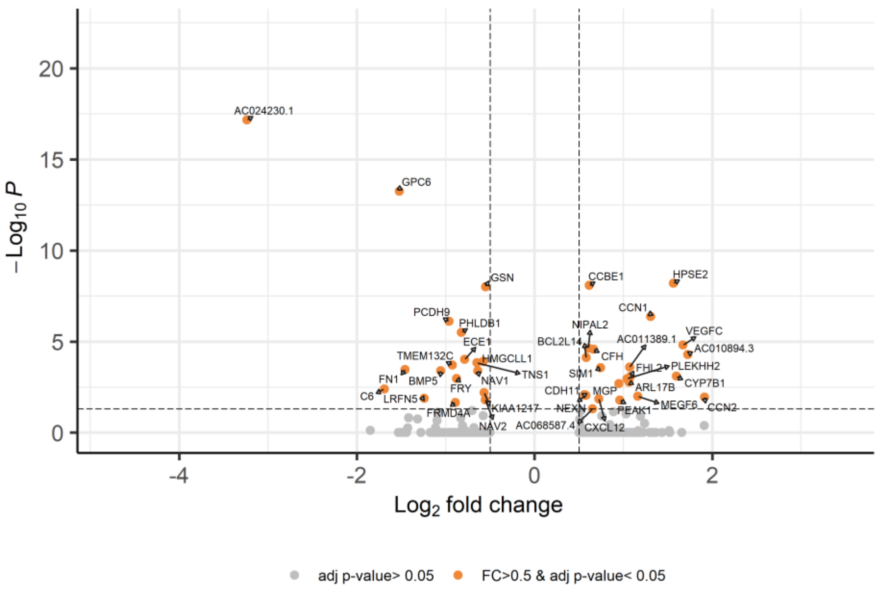

FAP_2 - KOA vs NO.KOA

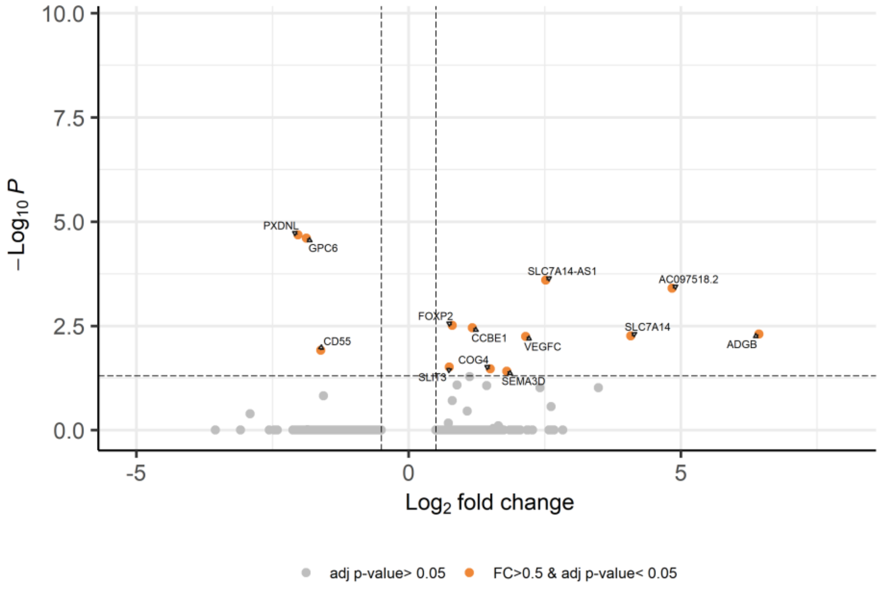

Figure S4: Volcano plot displaying differentially expressed genes (DEGs) in the progenitor cell sub-populations between KOA and NO.KOA group (No DEGs were obtained for FAP_3 and satellite cell population). Genes with a positive log fold change are increased in the KOA group. Dots marked in orange have a log2 fold change above +0.5 or -0.5 and an adjusted p-values<0.05. Dots marked in grey have an adjusted p-values>0.05

Supplementary Figure S5

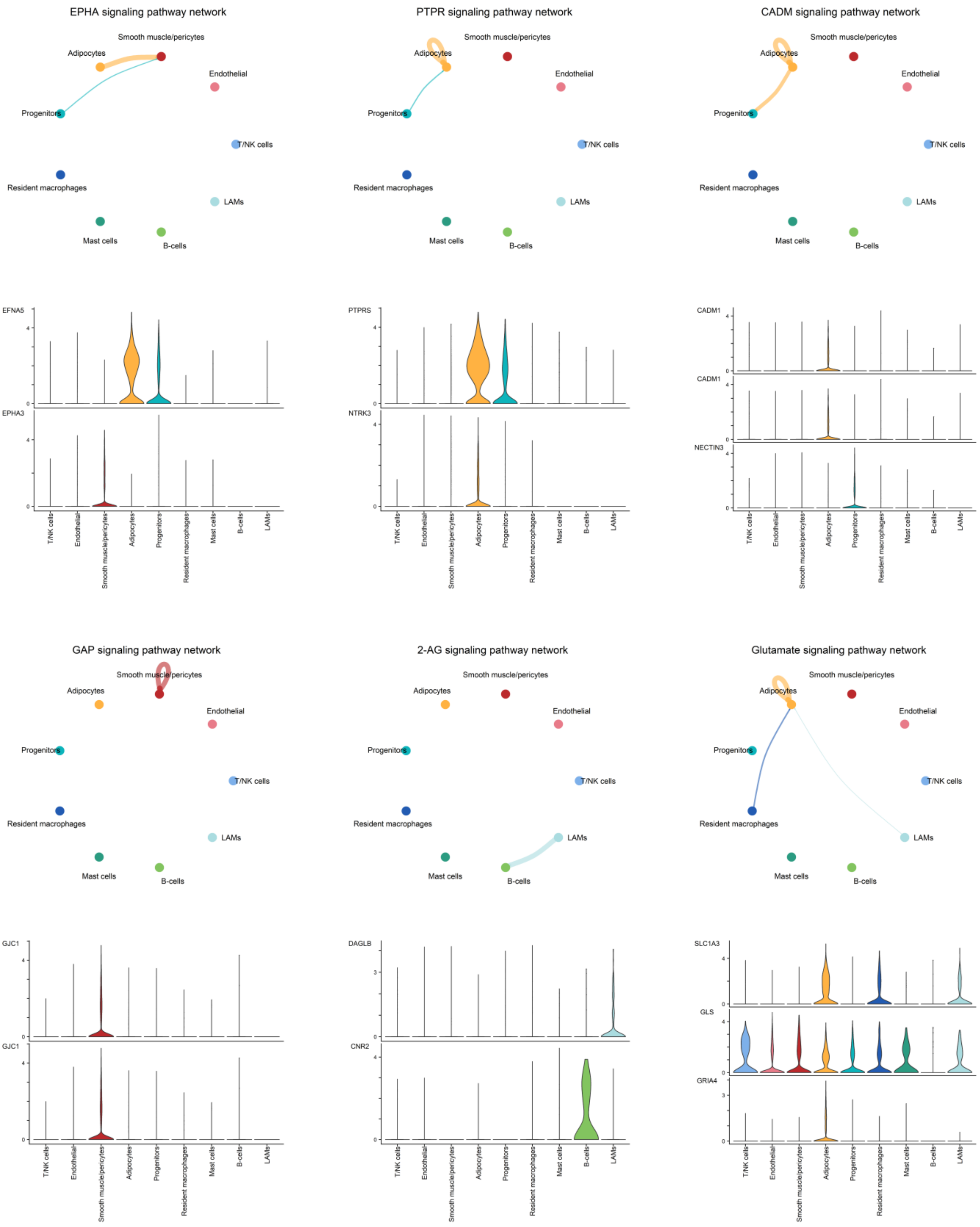

Figure S5: Circle and violin plots (matched vertically) of each additional signaling pathways enriched in the KOA group. In the circle plots the color of the line specifies which cell type the signal is coming from. The thickness of the line corresponds to the interaction strength. Thicker edge line indicates stronger signal.

Supplementary Figure S6

LEPTIN

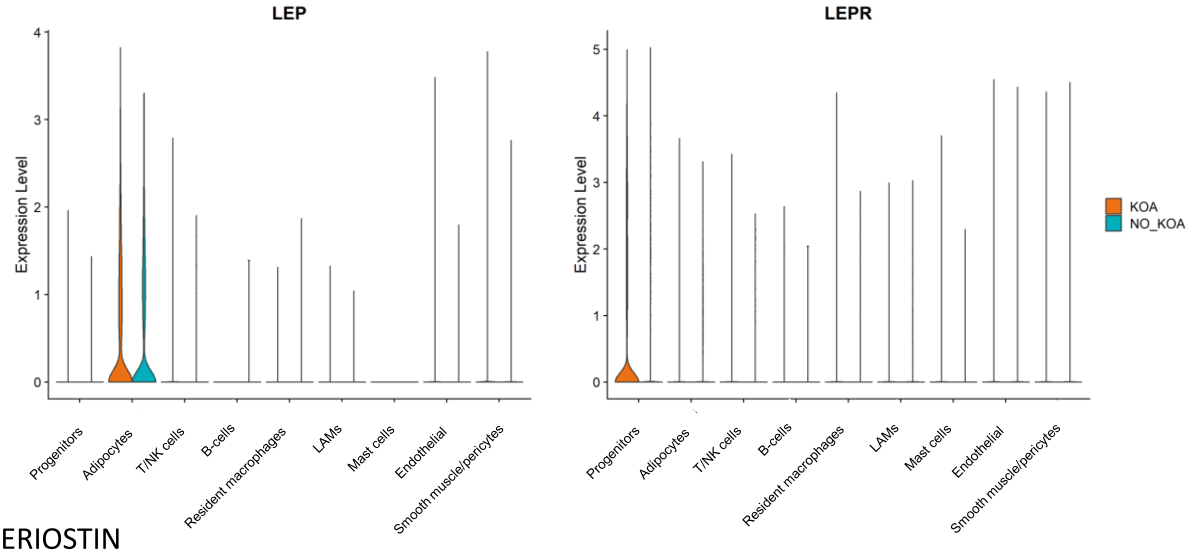

PERIOSTIN

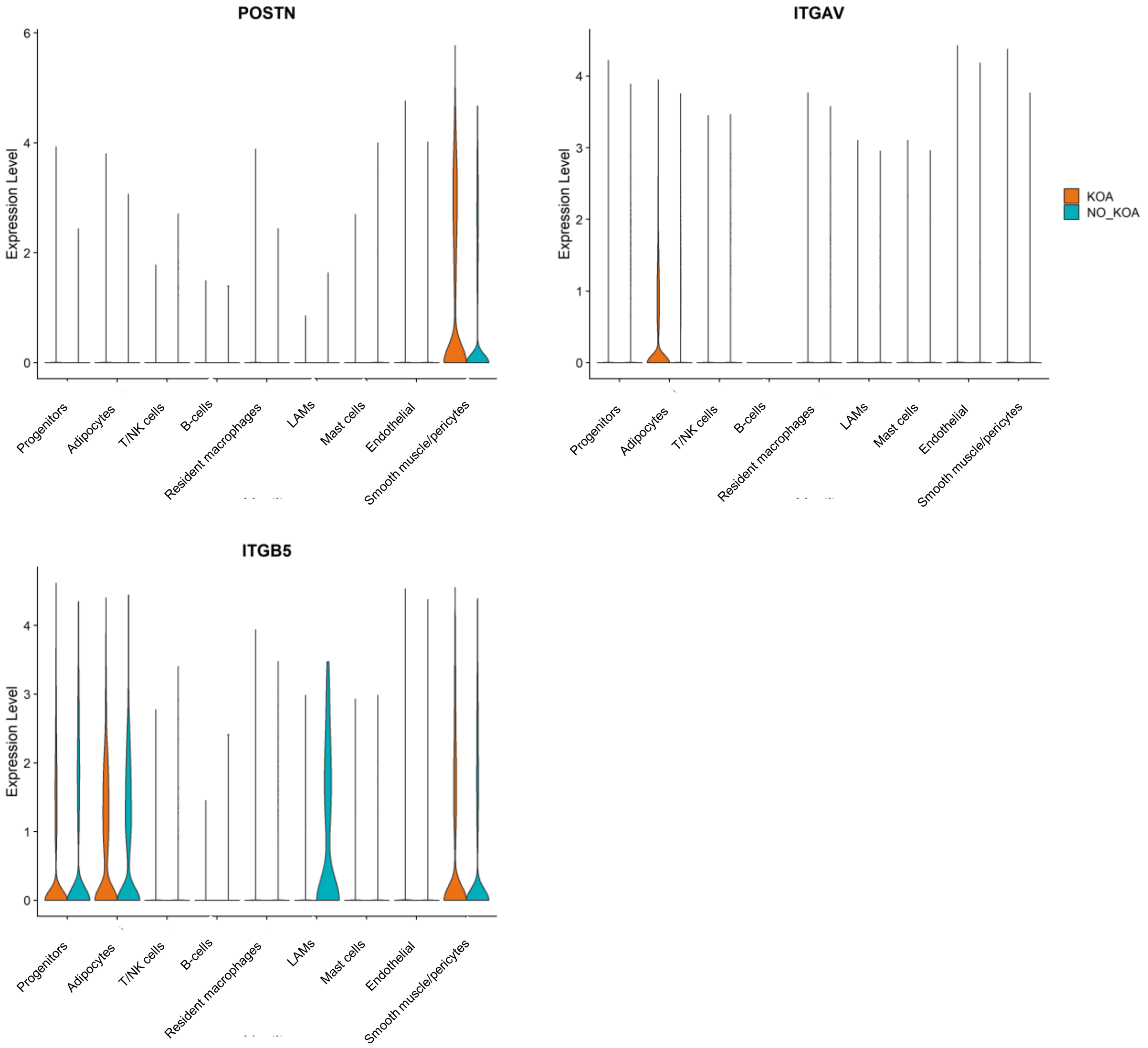

Figure S6: Violin plots showing the expression levels of receptor and ligands involved in each of the KOA-enriched signaling pathway split by the two groups.

Supplementary Figure S6 (continued)

EPHA

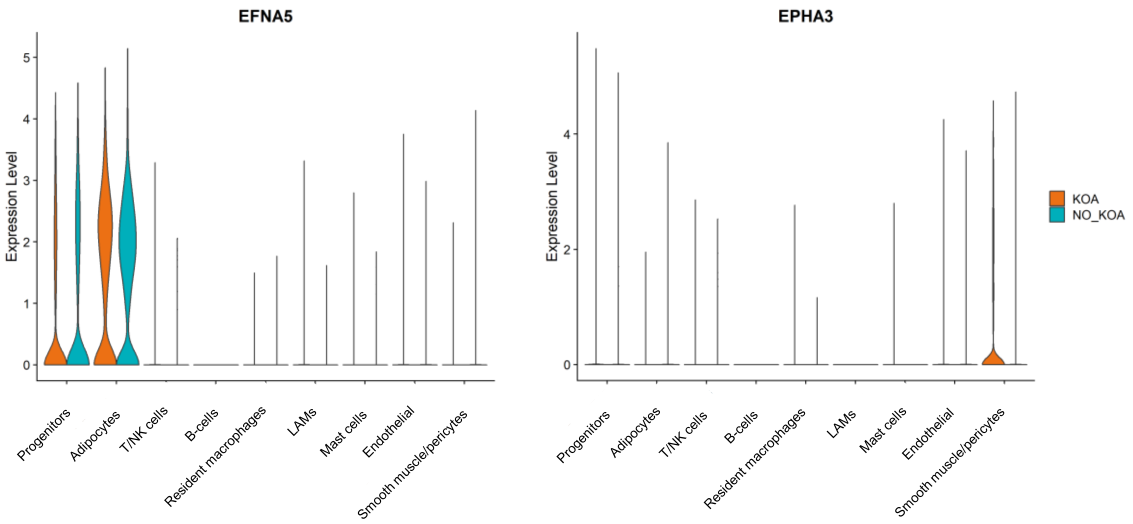

PTPR

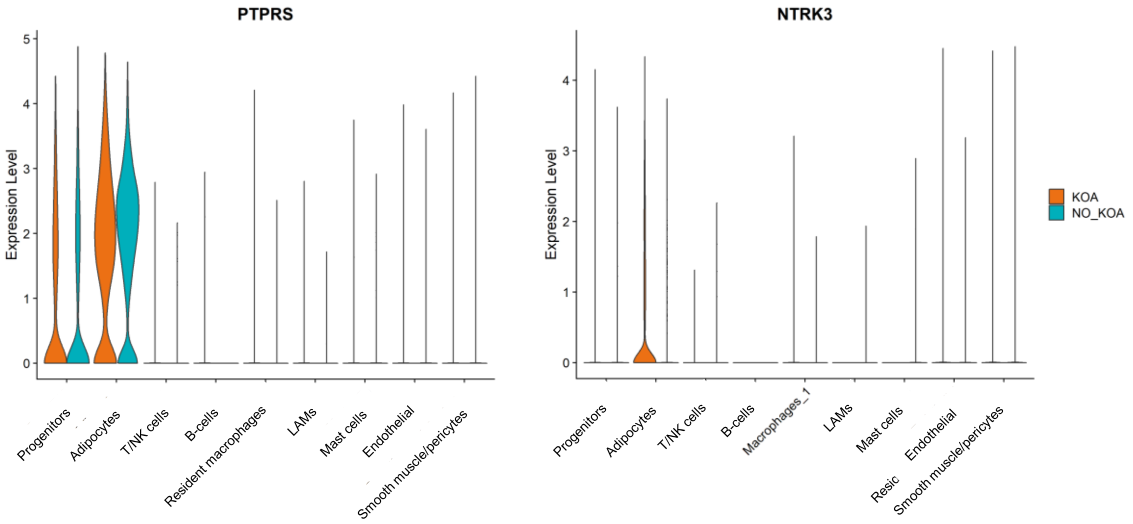

CADM1

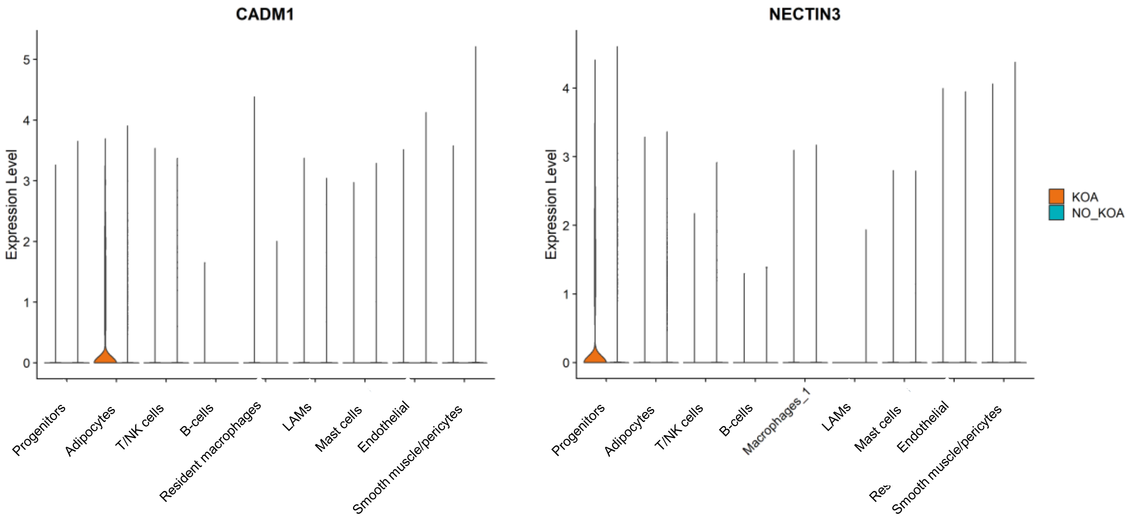

GAP

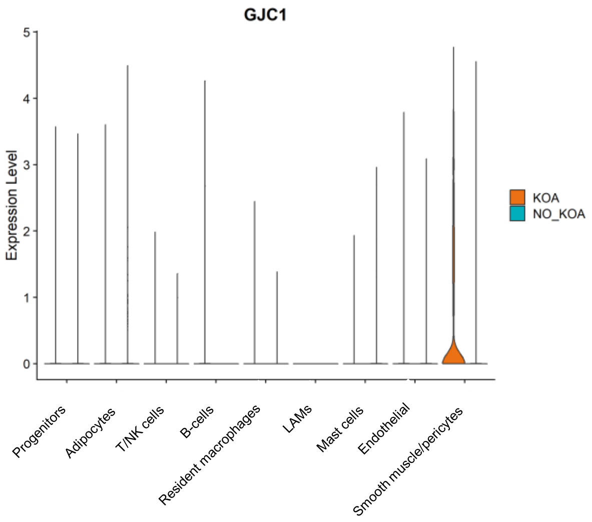

Supplementary Figure S6 (continued)

2-AG

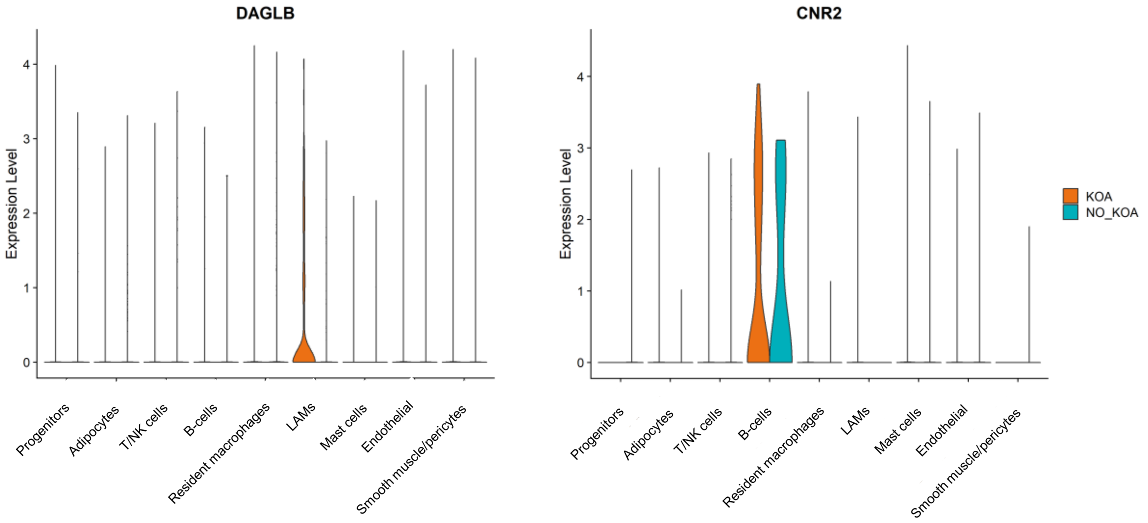

GLUTAMATE

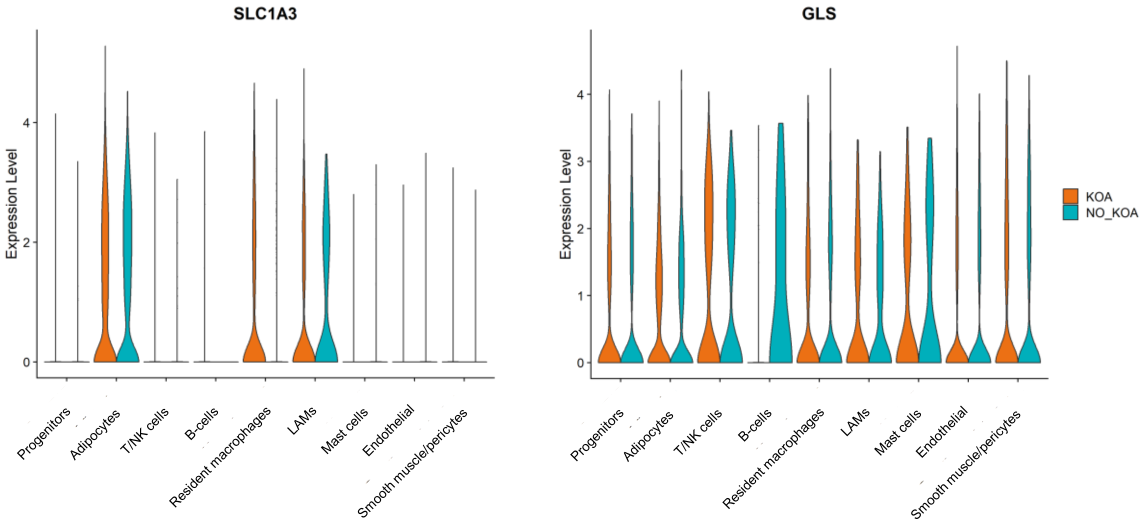

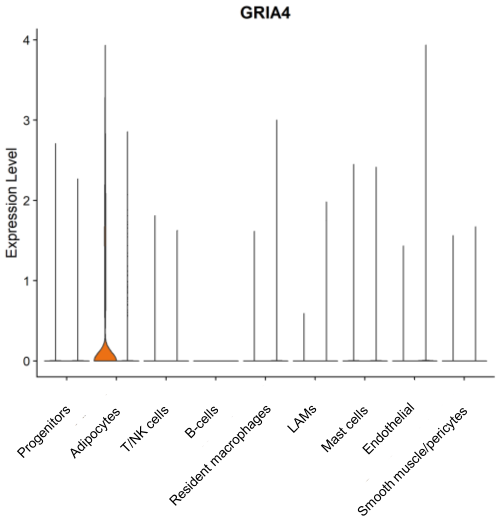

Supplementary Figure S7

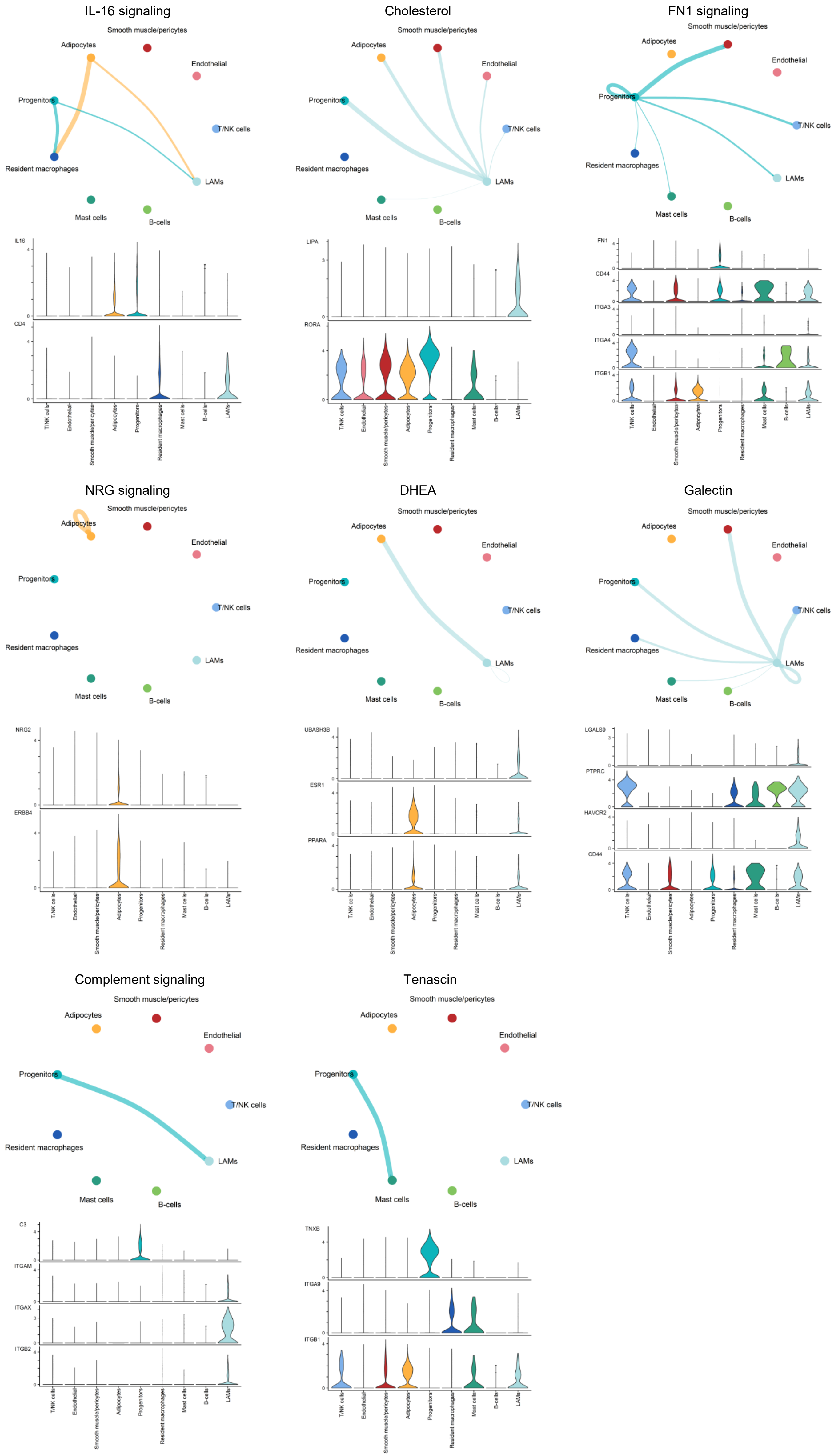

Figure S7: Circle and violin plots (matched vertically) of signaling pathways enriched in the NO.KOA group. In the circle plots the color of the line specifies which cell type the signal is coming from. The thickness of the line corresponds to the interaction strength. Thicker edge line indicates stronger signal.

Supplementary Figure S8

IL16

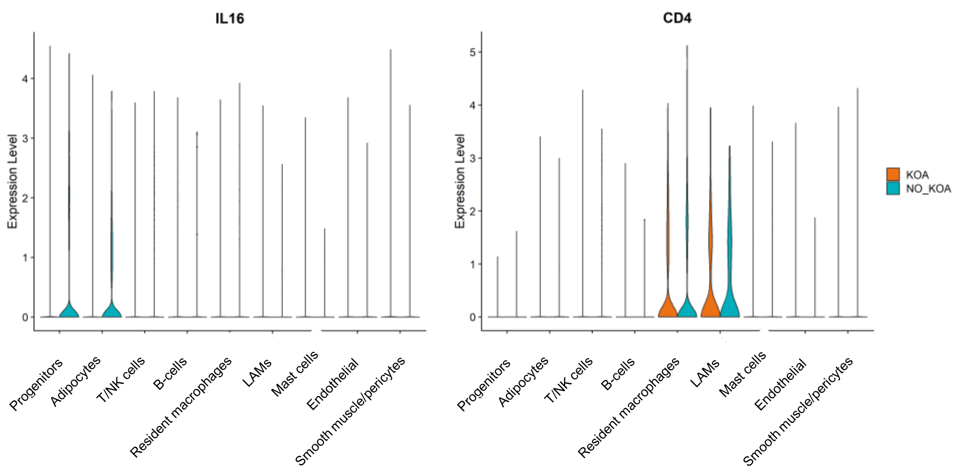

CHOLESTEROL

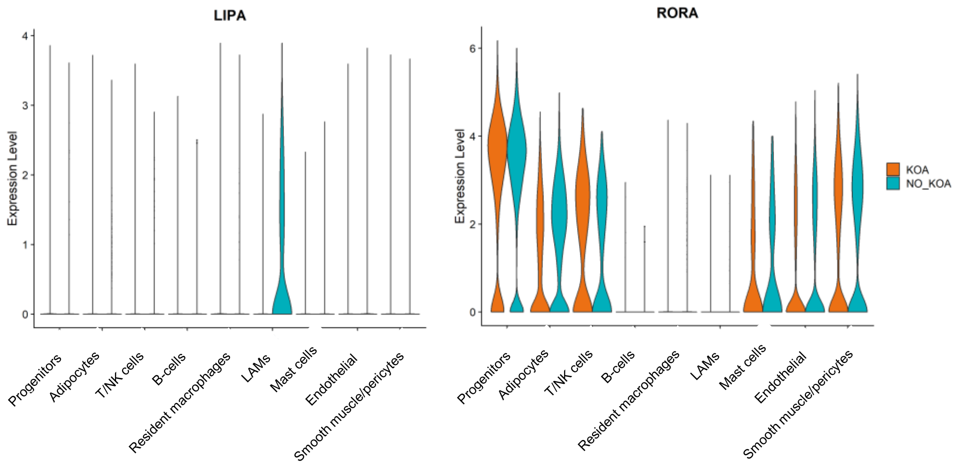

FN1

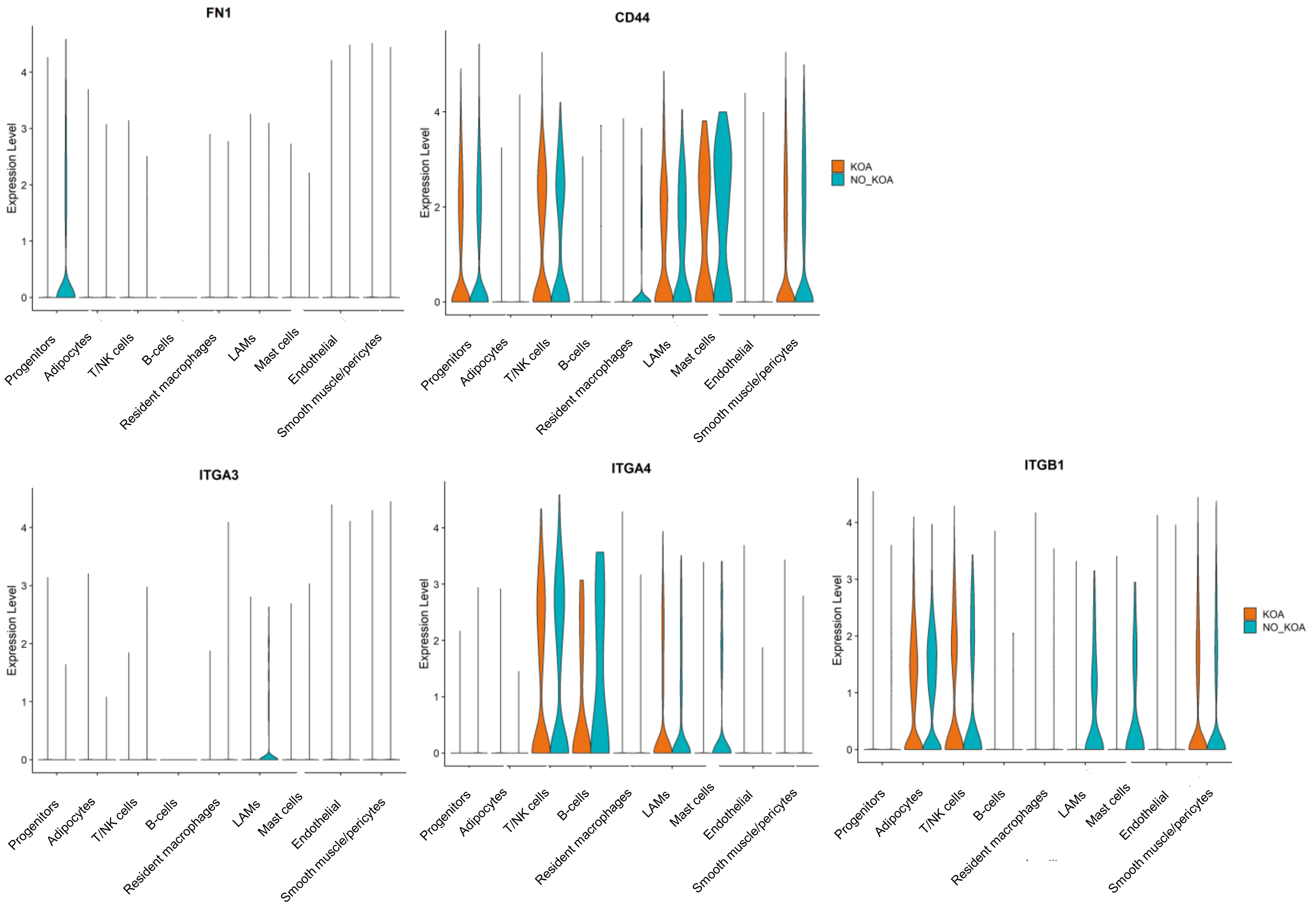

Figure S8: Violin plots showing the expression levels of receptor and ligands involved in each of the NO.KOA-enriched signaling pathways split by the two groups.

Supplementary Figure S8 (continued)

GALECTIN

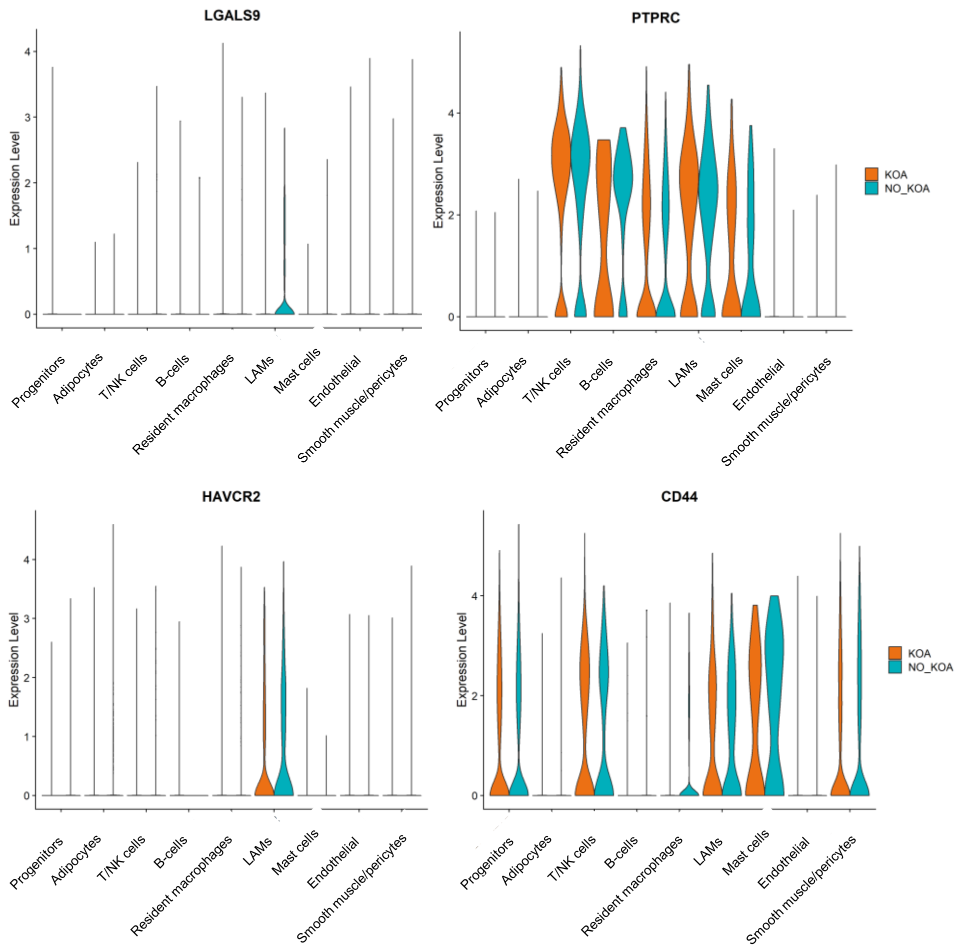

COMPLEMENT

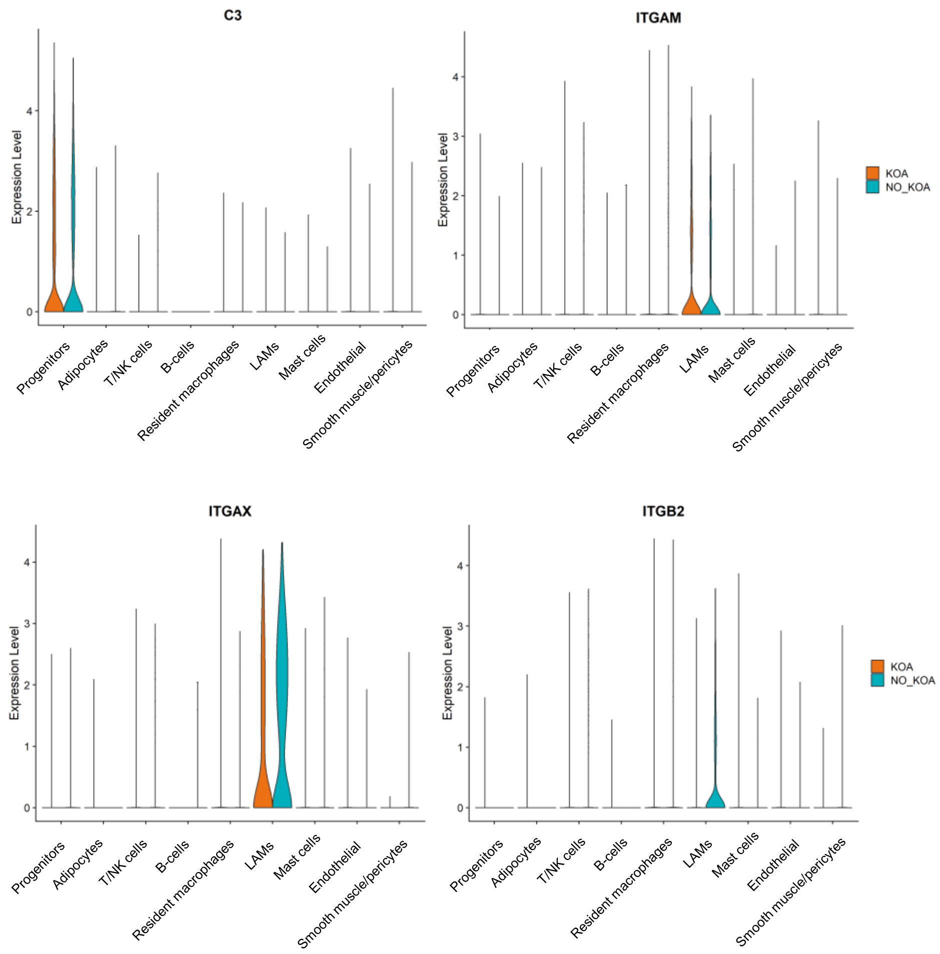

Supplementary Figure S8 (continued)

NRG

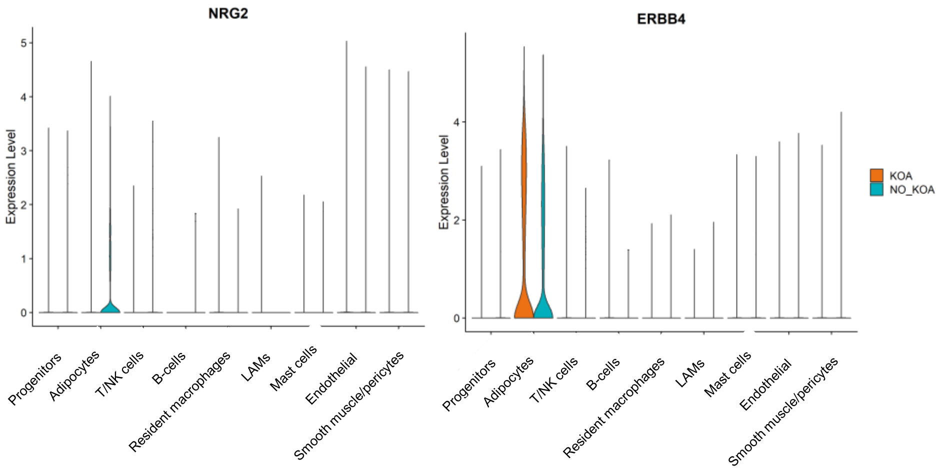

TENASCIN

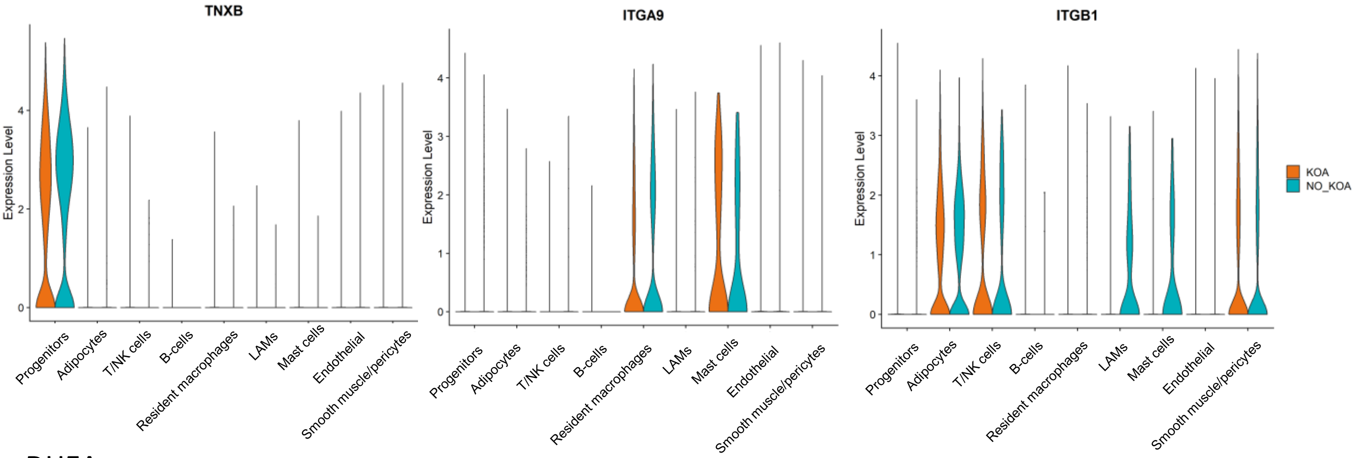

DHEA

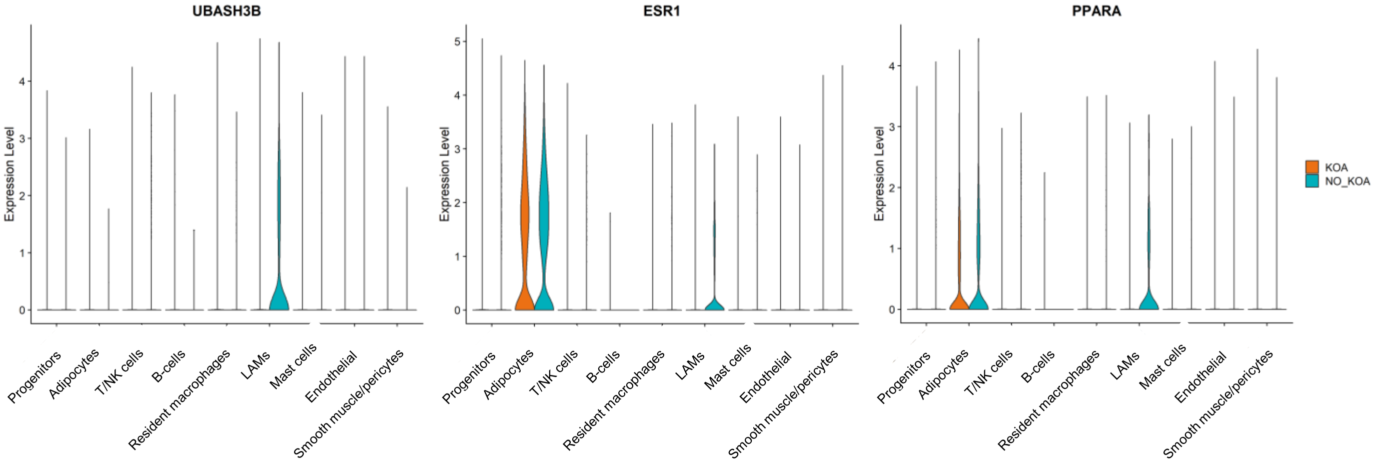

Supplementary Figure S9

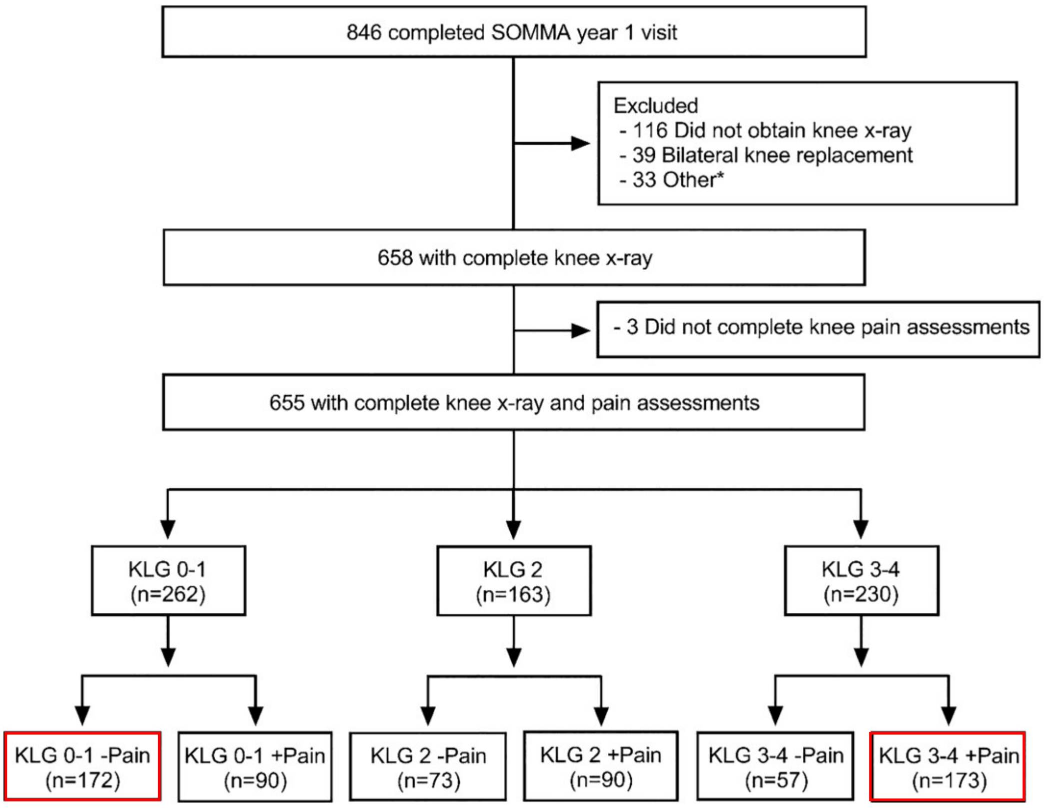

Figure S9: Study flowchart of the SOMMA KOA ancillary study presented in Distefano G, Harrison S, Lynch J, et al. *Skeletal Muscle Composition, Power, and Mitochondrial Energetics in Older Men and Women With Knee Osteoarthritis.* Arthritis Rheumatol. 2024;76(12):1764-1774. doi:10.1002/art.42953. Participants included in this study were all women selected from the boxes highlighted in red based on having a muscle biopsy enriched for IMAT.

Supplementary Figure S10

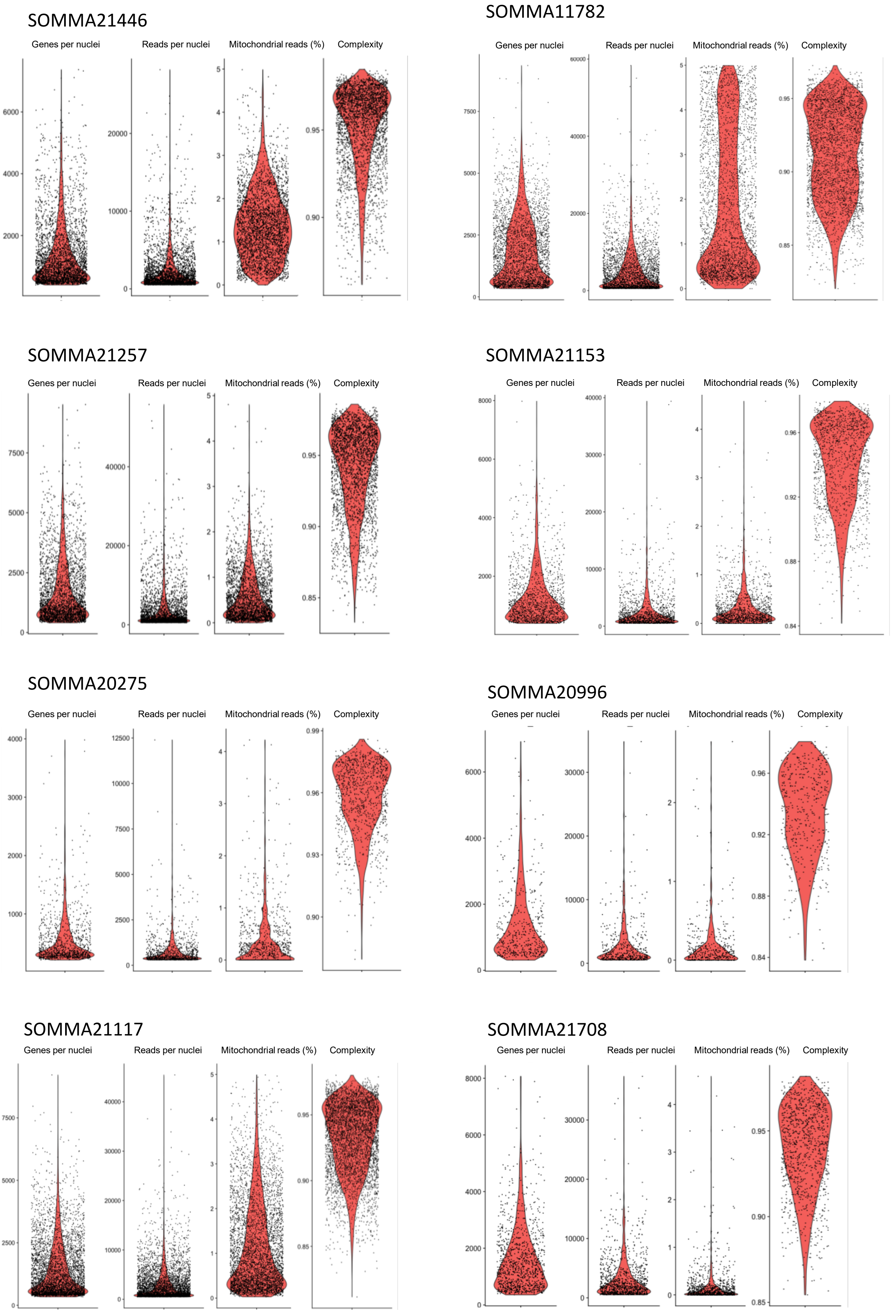

Figure S10: Violin plots depicting QC metrics for individual samples after filtering. Each dot is a nuclei.

Supplementary Figure S10 (continued)

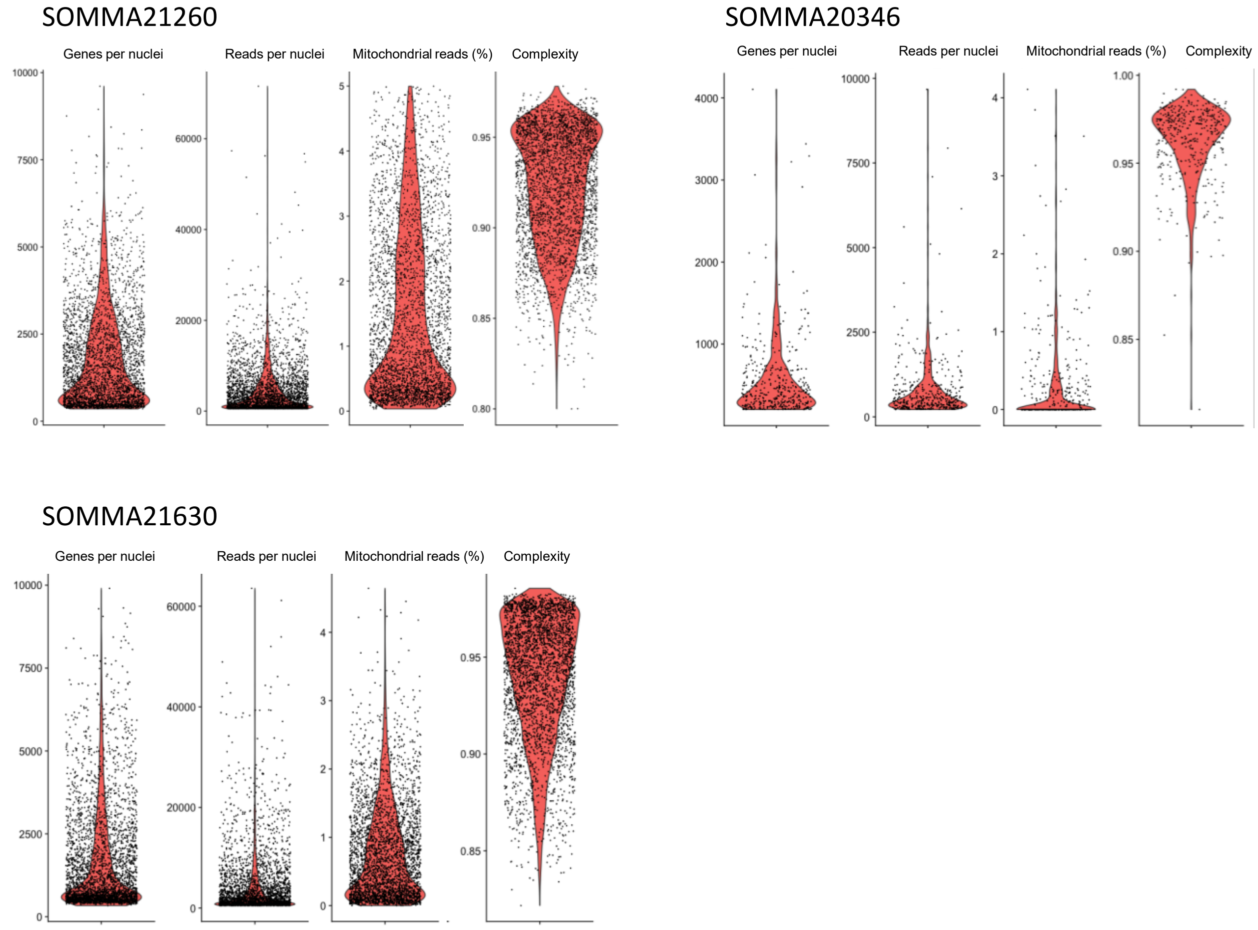

Supplementary Figure S11

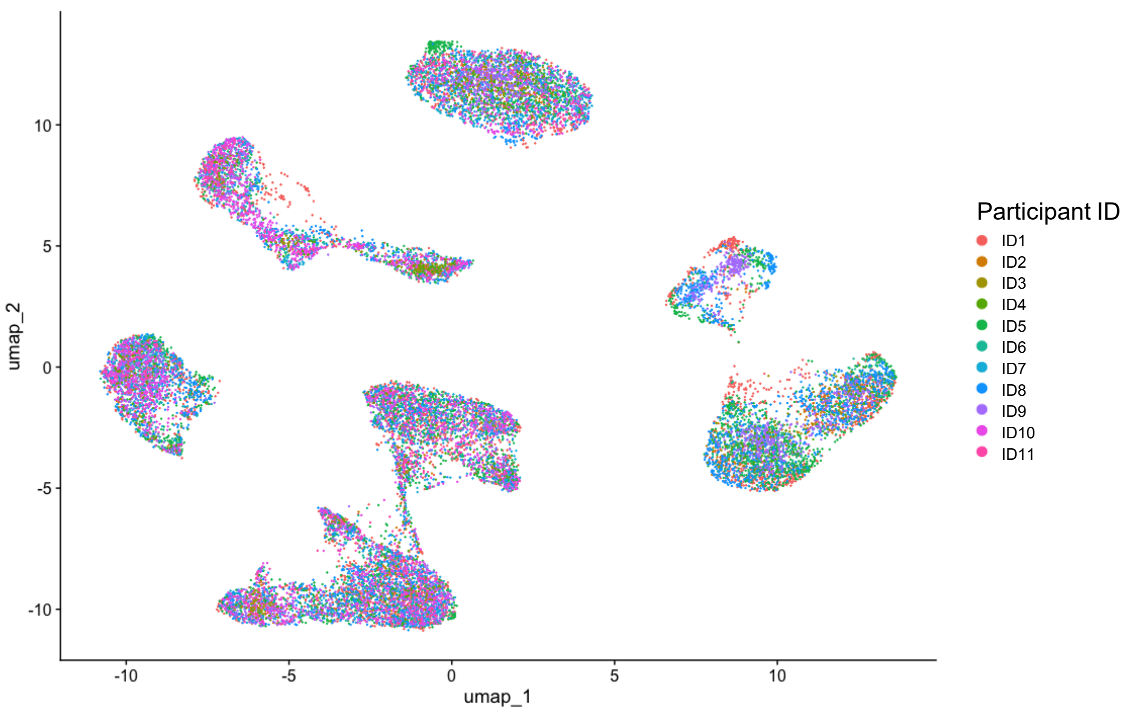

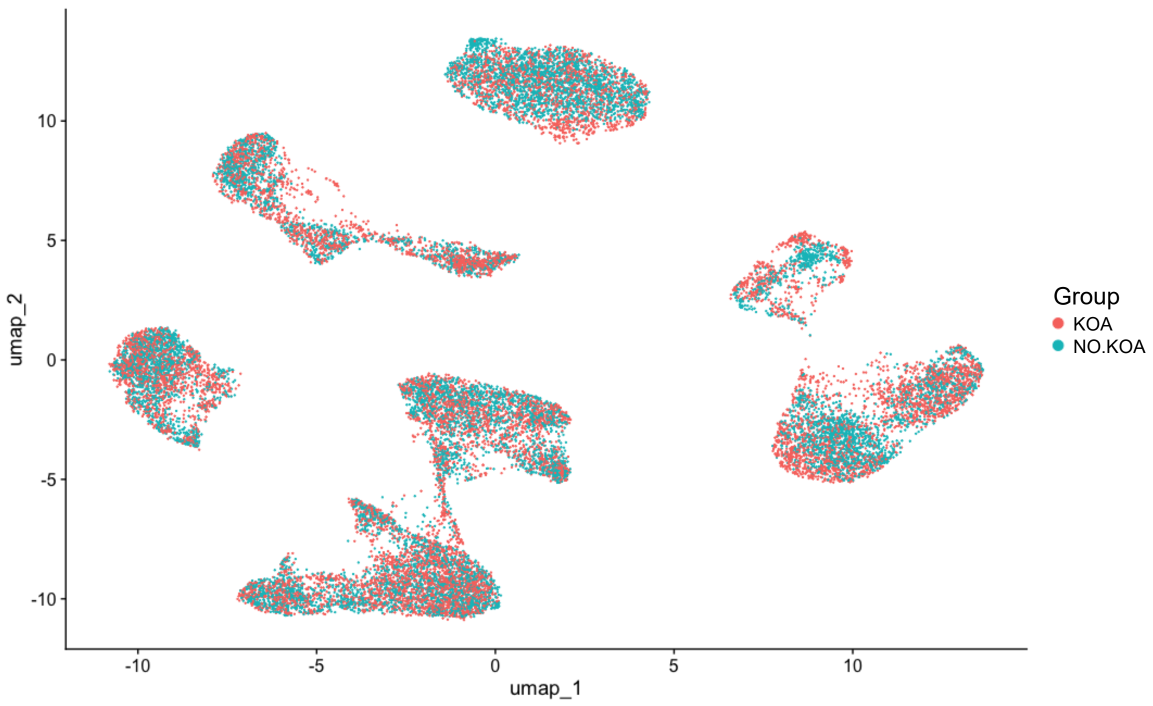

Figure S11: UMAP displaying clustering of nuclei according to Participant ID and Group after batch correction with harmony integration.

Supplementary Figure S12

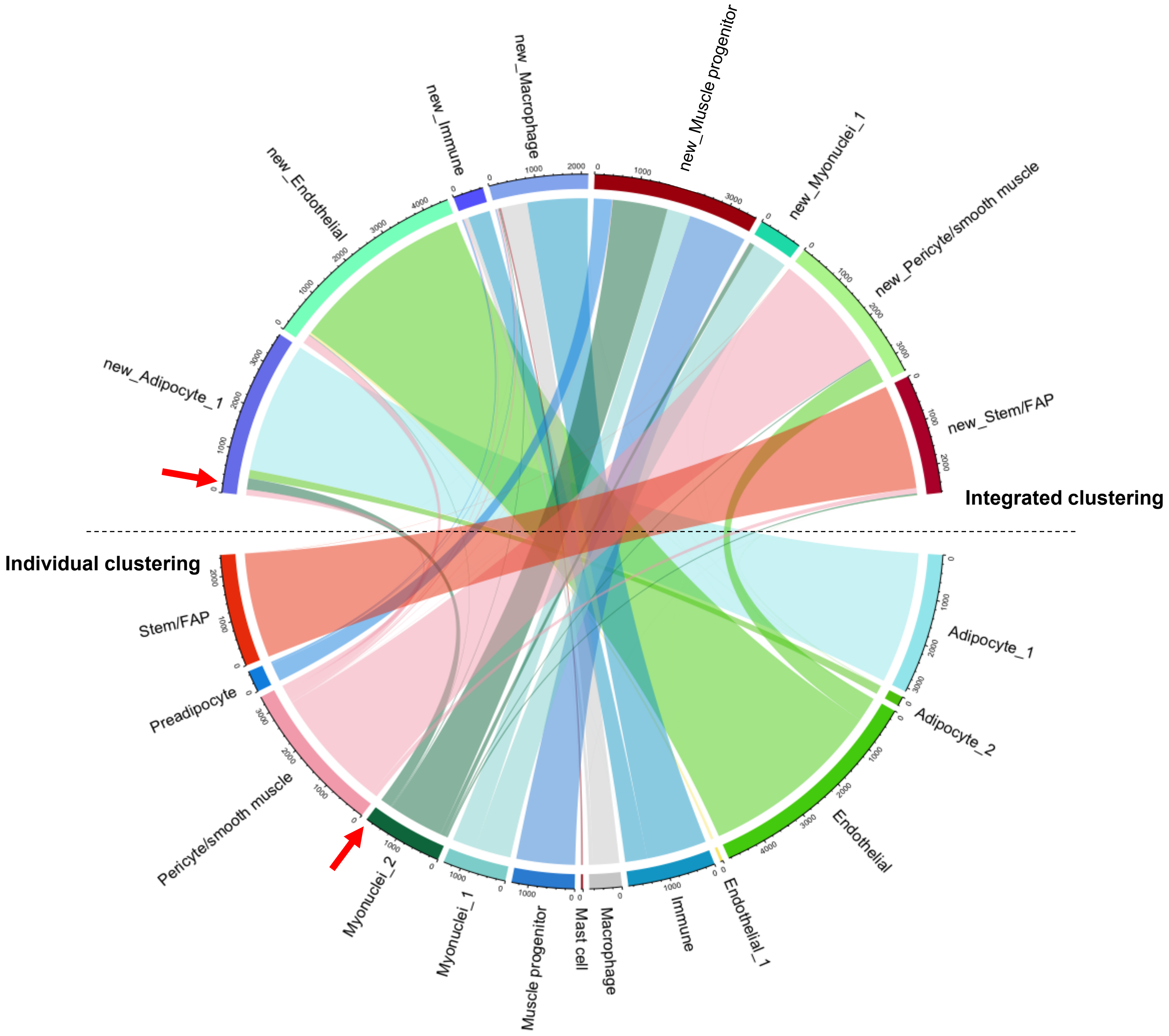

Figure S12: Circle plot displaying cell type annotation before and after harmony integration. The lower half of the circle indicates the cell type annotation each nuclei was categorized in when clustering the samples individually. The upper half of the circle indicates the new cell annotation clusters after integration of all 11 samples using harmony. The red arrows mark the Myonuclei population shifting to Adipocyte population after integration indicating poor quality of the nuclei. These nuclei were subsequently removed from dataset.

Supplementary Figure S13

A

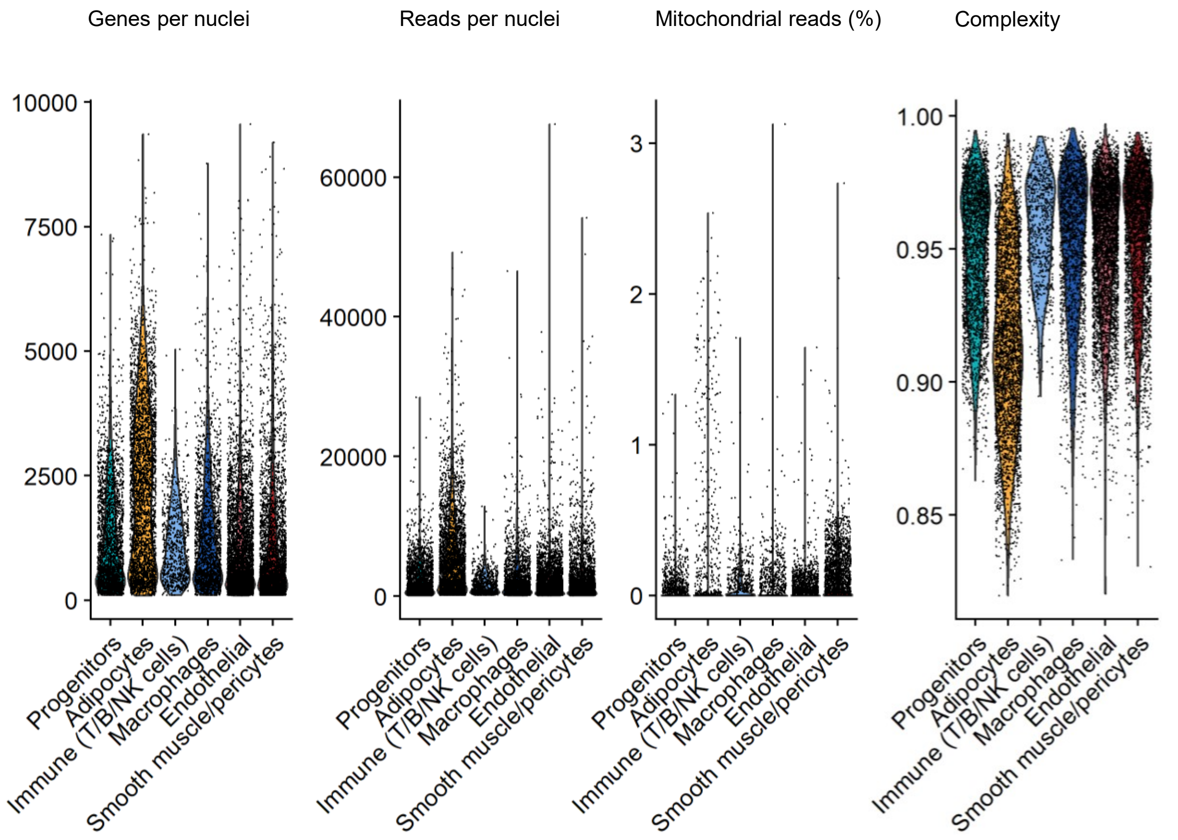

B

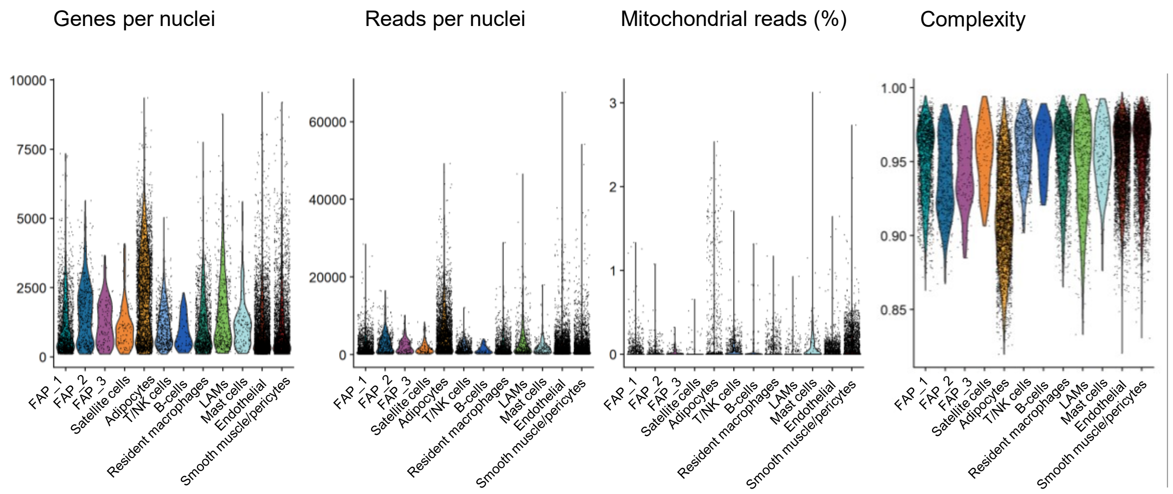

Figure S13: Violin plots depicting QC metrics for each of the major cell type populations **(A)** and subpopulation **(B)** after data integration. Each dot is a nuclei.
